# Zebrafish Heart Failure Models

**DOI:** 10.3389/fcell.2021.662583

**Published:** 2021-05-20

**Authors:** Suneeta Narumanchi, Hong Wang, Sanni Perttunen, Ilkka Tikkanen, Päivi Lakkisto, Jere Paavola

**Affiliations:** ^1^Unit of Cardiovascular Research, Minerva Foundation Institute for Medical Research, Biomedicum Helsinki, Helsinki, Finland; ^2^Abdominal Center Nephrology, University of Helsinki, Helsinki University Hospital, Helsinki, Finland; ^3^Department of Clinical Chemistry and Hematology, University of Helsinki, Helsinki University Hospital, Helsinki, Finland

**Keywords:** zebrafish, heart failure, cardiac remodeling, cardiac hypertrophy, cardiomyopathy

## Abstract

Heart failure causes significant morbidity and mortality worldwide. The understanding of heart failure pathomechanisms and options for treatment remain incomplete. Zebrafish has proven useful for modeling human heart diseases due to similarity of zebrafish and mammalian hearts, fast easily tractable development, and readily available genetic methods. Embryonic cardiac development is rapid and cardiac function is easy to observe and quantify. Reverse genetics, by using morpholinos and CRISPR-Cas9 to modulate gene function, make zebrafish a primary animal model for *in vivo* studies of candidate genes. Zebrafish are able to effectively regenerate their hearts following injury. However, less attention has been given to using zebrafish models to increase understanding of heart failure and cardiac remodeling, including cardiac hypertrophy and hyperplasia. Here we discuss using zebrafish to study heart failure and cardiac remodeling, and review zebrafish genetic, drug-induced and other heart failure models, discussing the advantages and weaknesses of using zebrafish to model human heart disease. Using zebrafish models will lead to insights on the pathomechanisms of heart failure, with the aim to ultimately provide novel therapies for the prevention and treatment of heart failure.

## Introduction

Heart disease is the number one cause of mortality and years of life lost globally (GBD 2016 Causes of Death Collaborators, 2017). Heart failure (HF) is defined as the heart’s inability to provide sufficient blood to meet the body’s needs. More than 1% of the adult population has HF, and the 5-year mortality is over 50% (Bleumink et al., 2004). Due to aging populations, this severe clinical syndrome is becoming increasingly prevalent. By 2030, almost 3% of the population is predicted to have HF (Virani et al., 2020).

Coronary artery disease and hypertension account for over 80% of the etiology of HF. Other important etiologies include valvular heart diseases, cardiomyopathies (CMs), myocarditis, and inflammatory heart diseases. Dilated cardiomyopathy (DCM) and hypertrophic cardiomyopathy (HCM) are the most prevalent non-ischemic CMs, with estimated prevalence of >0.4% and >0.2%, respectively (McKenna and Judge, 2021). More rare causes of HF include congenital heart diseases, arrhythmias, cardiotoxicity, infections, high-output HF, right-sided HF, and pericarditis (Ponikowski et al., 2016).

Over the past couple of decades, zebrafish has emerged as a powerful vertebrate model for studying heart diseases. It is increasingly used to study development, anatomy, and physiology by genetic, pharmacological, and numerous other experimental techniques. The advantages of using zebrafish are clear (Table 1A). Zebrafish are used for high-throughput drug screening and for assessing drug efficacy and toxicity (Gut et al., 2017). The zebrafish genome is fully sequenced and readily available (Howe et al., 2013). Humans and zebrafish are genetically similar, with the great majority of genes having orthologs with significant similarity at the protein level (Barbazuk et al., 2000). This enables the use of targeted mutagenesis techniques for modeling human genetic diseases (Arnaout et al., 2007).

**TABLE 1:** Advantages and disadvantages of using zebrafish for modeling human diseases in general **(A)** and specifically for cardiac diseases **(B)**.

A	Advantages (general)	Disadvantages (general)
	• Ease, speed, and affordability of maintenance and breeding	• Duplicated genome
	• Small size and optical transparency	• Relative unavailability of antibodies
	• Rapid development	
	• High-throughput drug screening	
	• Ease and availability of genetic manipulation	

**B**	**Advantages (cardiac)**	**Disadvantages (cardiac)**

	• Rapid development: heartbeat begins already at 24 h post-fertilization	• Two-chambered heart lacking pulmonary circulation
	• Blood circulation not needed for development in the first week of life, possible to study severe cardiovascular phenotypes	• Hemodynamics: low central venous pressure, ventricular filling relies mainly on atrial contraction
	• Conserved fundamentals of excitation-contraction coupling	• Electrophysiology: differences in inward currents, sodium current is lower and calcium current higher
	• Electrophysiology: similar heart rate and action potential duration and morphology	• Electrophysiology: lack of transient outward potassium current and slow component of the delayed rectifier current
	• Cardiac regeneration following injury	• Minor role of SR calcium stores for muscle contraction and triggered arrhythmias, negative FFR
		• Lack of sarcolemmal T-tubules in cardiomyocytes
		• Scarcity of chronic cardiac fibrosis and cardiac fat
		• Tiny size of the embryonic heart complicates handling and experimentation with staining, qPCR etc.
		• Small size and trabeculation of the adult heart muscle complicates imaging and quantification of cardiac function with echocardiography

The technique of anti-sense morpholino oligonucleotides (MOs) for targeted knockdown of genes has been used for over two decades (Nasevicius and Ekker, 2000). MOs must be distinguished from the following truly genetic methods that produce heritable changes, for MO knockdown is transient, as the anti-mRNAs injected into fertilized eggs gradually dilute within a few days (Nasevicius and Ekker, 2000). In recent years, several genetic methods have been used for gene-specific targeting. These include engineered nucleases, such as the zinc-finger nucleases (ZFNs) (Doyon et al., 2008; Meng et al., 2008) and the transcription activator-like effector nucleases (TALENs) (Huang et al., 2011; Sander et al., 2011), and most recently, the CRISPR-Cas9 (Hwang et al., 2013). CRISPRs have recently been established as a powerful reverse genetic screening strategy in the zebrafish (Shah et al., 2015).

Here we first briefly review early developmental mutants discovered in forward genetic mutagenesis screens, as they demonstrated the value of investigating cardiac phenotypes in embryonic zebrafish, and paved way for the increasing number of studies using reverse genetic techniques for modeling cardiovascular disease in zebrafish. These developmental mutants are not reviewed in detail, as they are not primarily for modeling HF. We review both embryonic and adult zebrafish models for studying cardiac remodeling and impaired cardiac function, grouping these into main groups of genetic and drug-induced models, and also examine other HF models. As the etiologies of HF range from fully congenital to fully acquired and everything in between, we seek to include examples from this vast range, focusing on the suitability of zebrafish models for investigating candidate genes and genetic factors predisposing to HF. We discuss the genetic and physiological suitability of using zebrafish to model human HF. For clarity, here we arbitrarily call all zebrafish younger than 7 days post fertilization (dpf) as “embryonic,” including larval zebrafish older than 3 dpf.

## Defining Heart Failure in Zebrafish

**FIGURE 1:**
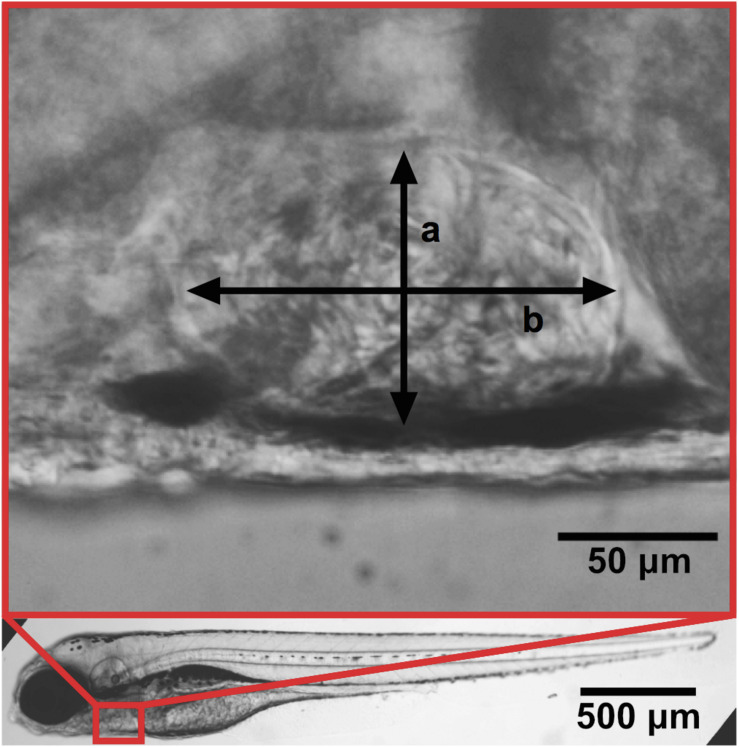
Zebrafish at 4 dpf with a zoom-in of the ventricle. Ventricular width a and length b. Adapted with permission from Paavola et al. (2020).

First, it is vital to define what is meant by HF in zebrafish embryos and adults, and how cardiac function is measured and quantified. In embryonic zebrafish assessing cardiac size and function is easy under a normal stereo-microscope due to optical translucency. Adult zebrafish require echocardiography, which is technically and financially more challenging. Both embryonic and adult fish require anesthesia for the imaging. A combination of the following factors should be used when evaluating cardiac function:

(a) Ventricular size. Increased ventricular size is associated with HF.
In embryos ventricular area or at least short and long axes of the ventricle measured at the end of systole and at the end of diastole under a normal stereo-microscope (Figure 1).

**FIGURE 2:**
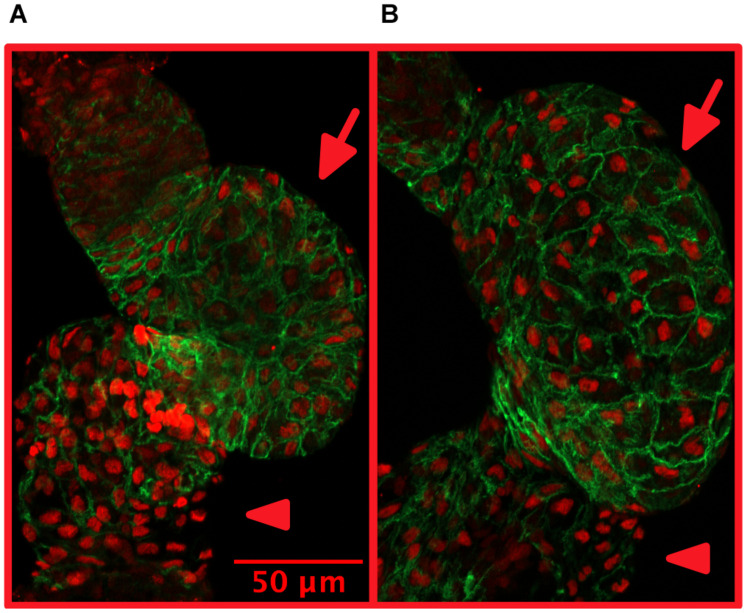
Treatment with isoproterenol increases cardiomyocyte size. 4 dpf zebrafish without **(A)** and with **(B)** 300 μM isoproterenol-treatment 2–4 dpf. Staining with Mef-2 antibody (red) to identify cardiomyocyte nuclei and with ZN-5 antibody (green) to delineate cardiomyocyte cell borders. Ventricles marked with arrows and atria marked with arrowheads. Adapted with permission from Paavola et al. (2020).

In adult zebrafish, cardiac ultrasound (echocardiography) is used (Wang et al., 2017).
More broadly, cardiac structure at the organ-level as a whole is important to assess already during *in vivo* video-microscopy and echocardiography. This will then direct following steps into possible biochemical experiments and microscopic imaging.
Growth of the heart muscle may involve growth of cell size (hypertrophy) or growth of cardiomyocyte number due to proliferation (hyperplasia). Size and number (in embryos) of cardiomyocytes may be quantified from dissected and stained zebrafish hearts (Figure 2).

(b) Cardiac contractility and relaxation. Impaired contractility and prolonged relaxation are associated with HF.
Cardiac function may be quantified in several ways and the same parameters can be used in embryonic and adult fish. Common ways of measuring contractility are ejection fraction and fractional shortening (Fink et al., 2009). Fractional shortening may be measured 1- (diameter), 2- (area), or 3- (volume) dimensionally. In adult zebrafish, we found fractional volume shortening to be the most reliable method (Narumanchi et al., 2019). From the same fast frame-rate recordings, cardiac relaxation time may be measured (Paavola et al., 2020).
In embryonic zebrafish, stroke volume may also be quantified with measurement of erythrocyte flow velocity in the aorta (Paavola et al., 2013). When measurements of ventricular size are challenging, complicated by e.g., pericardial edema (see below), blood flow measurement may be more reliable.
Additionally, valvular function is important to assess to rule out flow-limiting stenosis or backflow.
(c) Edema. Increased edema is associated with HF.
Pericardial edema is easy to quantify in embryonic zebrafish (Paavola et al., 2020), however on its own it is a very non-specific finding. To our knowledge, pericardial or peripheral edema remain largely unquantified in adult zebrafish. However, accurate weighing is one way to follow fluid build-up.
(d) Rhythm. Cardiac arrhythmias and conduction delays are associated with HF.
Zebrafish are prone to changes in heart rate and development of atrioventricular conduction block (Milan et al., 2003; Paavola et al., 2013; Zhu et al., 2014). Various methods to investigate cardiac rhythm and conductance in embryonic and adult zebrafish have been established (Santoso et al., 2020). Electrocardiogram (ECG) remains the gold standard in adult zebrafish (Milan et al., 2006).
(e) Biochemical HF markers. Atrial natriuretic peptide (ANP) and brain natriuretic peptide (BNP) are the most established biomarkers that show increased expression levels in HF (Becker et al., 2012; Shi et al., 2017).

As in humans, a combination of the above-mentioned factors may indicate HF. For concluding HF in zebrafish, abnormal cardiac structure and/or function (impaired blood flow, contractility or relaxation) must be shown. In addition to the above-mentioned factors, the interpretation as HF is further supported by the zebrafish showing rapid breathing, exercise intolerance, and increased mortality (Wang et al., 2011).

Additionally, numerous less used/available methods for quantifying heart size and function have been reported, including magnetic resonance imaging (Koth et al., 2017; Merrifield et al., 2017). More broad review of experimental techniques such as optogenetics (Arrenberg et al., 2010), voltage mapping and calcium imaging is beyond the scope of this article (Genge et al., 2016; Lin et al., 2020).

## Genetic Heart Failure Models

Several zebrafish models of cardiac hypertrophy, hyperplasia, dysfunction, and remodeling have been established by genetic manipulation (Table 2) or by drug treatment (Table 3).

**TABLE 2:** Genetic models of heart failure.

Gene	Embryo/Adult	Knock-out (KO)/Morpholino/Other	Main Phenotype	References
*bag3*	Both	Morpholino and KO	Cardiac dysfunction	Norton et al., 2011; Ding et al., 2019
*band3*	Adult	KO	hypertrophy and hyperplasia	Sun et al., 2009
*cmlc1*	Adult	KO	Arrhythmia	Orr et al., 2016
*dco^*s*226^*	Embryo	KO	Conduction defect	Chi et al., 2010
*erbb2*	Both	KO	Hypertrophy, cardiac dysfunction	Reischauer et al., 2014; Fleming et al., 2018
*gtpbp3*	Both	KO	Hypertrophy	Chen et al., 2019
*heg1*	Embryo	KO	Cardiac dysfunction	Lu et al., 2020
*hhatla*	Embryo	Morpholino	Hypertrophy, cardiac dysfunction	Shi et al., 2020b
*ilk*	Embryo	KO	Cardiac dysfunction	Bendig et al., 2006
*jag2b*	Both	Genetic ablation	Hypertrophy	Abdul-Wajid et al., 2018
*lamp2*	Adult	KO	Hypertrophy, cardiac dysfunction	Dvornikov et al., 2019
*lmcd1, tns1*	Embryo	Morpholino	Valvular heart defect	Dina et al., 2015
*lrrc10*	Embryo	Morpholino	Hypertrophy	Kim et al., 2007
*lztr1*	Adult	KO	Hypertrophy	Nakagama et al., 2020
*mcu*	Both	KO	Cardiac dysfunction, arrhythmia	Langenbacher et al., 2020
*mybpc3*	Embryo	Morpholino	Hypertrophy	Chen et al., 2013
*myh6*	Adult	KO	Hyperplasia	Sarantis et al., 2019
*ndufa7*	Embryo	Morpholino	Hypertrophy, cardiac dysfunction	Shi et al., 2020a
*pkd2*	Embryo	Morpholino	Cardiac dysfunction, AV-block	Paavola et al., 2013
*ptpn11*	Embryo	mRNA injections	left-right asymmetry	Bonetti et al., 2014
*rbfox1*	Embryo	Morpholino	Cardiac dysfunction	Gao et al., 2016
*sept7b*	Embryo	Morpholino	Cardiac dysfunction	Dash et al., 2017
*tnnt2*	Embryo	KO and morpholino	Cardiac dysfunction	Sehnert et al., 2002; Becker et al., 2011
*trim55*	Embryo	KO	Cardiac dysfunction	Heliste et al., 2020
*ttn*	Embryo	KO	Cardiac dysfunction	Xu et al., 2002
*vclb*	Adult	KO	Hyperplasia	Cheng et al., 2016
*vegfaa*	Adult	Overexpression	Hyperplasia	Karra et al., 2018
*vezf1*	Embryo	Morpholino	Attenuation of cardiac growth	Paavola et al., 2020
*z-usmg5*	Embryo	Morpholino	Cardiac dysfunction	Nagata et al., 2017
*kif20a*	Embryo	Morpholino	Restrictive cardiomyopathy	Louw et al., 2018
*Plakoglobin*	Until 3 months of age	2057del2 mutant	Arrhythmogenic right ventricular cardiomyopathy	Asimaki et al., 2014
*nnt*	Embryo	Morpholino	Left ventricular non-compaction cardiomyopathy	Bainbridge et al., 2015

**TABLE 3:** Drug-induced models of heart failure.

Drug	Embryo/Adult	Main Phenotype	References
Aristolochic acid (AA)	Embryo	Cardiac dysfunction	Huang et al., 2007, 2013
Benzo(a)pyrene (Bap)	Both	Hypertrophy	Huang et al., 2014
Doxorubicin	Embryo	Cardiac dysfunction	Liu et al., 2014
Isoproterenol (ISO)	Both	Cardiac dysfunction	Kossack et al., 2017
Phenyl hydrazine hydrochloride (PHZ)	Adult	Hypertrophy and hyperplasia	Ernens et al., 2018
Phenylephrine (PE)	Adult	Hypertrophy and hyperplasia	Romano and Ceci, 2020
Streptozocin	Adult	Diabetic cardiomyopathy	Wang et al., 2020
Terfenadine	Embryo	Cardiac dysfunction, AV-block	Gu et al., 2017
Tolterodine	Embryo	Arrhythmia	Burczyk et al., 2019
Verapamil	Embryo	Cardiac dysfunction	Zhu et al., 2018

Cardiac hypertrophy is a common adaptive change to pathological processes leading to cardiac dysfunction and failure. With currently available genetic tools to modify gene expression in zebrafish, several molecular pathways have been associated with development of cardiomegaly and cardiac dysfunction. The list of reported genetic HF models in zebrafish, some of which are briefly described below and in Table 2, is constantly expanding.

The first zebrafish genetic models of hypertrophy/hyperplasia and cardiac dysfunction, *silent heart (sih)* and *pickwick^*m*171^* (*pik^*m*171^*), having mutations in *tnnt2* and *ttn*, respectively, were reported in Sehnert et al. (2002); Xu et al. (2002). *tnnt2* encodes cardiac Troponin T (cTNT), a contractile protein involved in cardiac muscle contraction. Mutations in *tnnt2* cause familial HCM (Thierfelder et al., 1994) and DCM (Kamisago et al., 2000). *sih* mutant hearts are non-contractile, the embryos die at 7 dpf when circulation becomes necessary. Pericardial edema is observed in embryos and sarcomere assembly is disturbed (Sehnert et al., 2002). *tnnt2* knockdown zebrafish embryos reveal sarcomeric disarray and cardiac hyperplasia (Becker et al., 2011). *ttn* encodes Titin, a large sarcomeric protein. Also mutations in *ttn* cause DCM and HCM (Gigli et al., 2016). *ttn* mutant hearts contract poorly, edema develops and the embryos die. The absence of titin results in failure of sarcomeric assembly (Xu et al., 2002). These first models, identified from forward genetic mutagenesis screens, demonstrated the value of embryonic zebrafish developing without functional circulation for the study of severe cardiac phenotypes.

Another sarcomere protein, myosin binding protein C (MyBP-C) maintains structural integrity of the sarcomere and regulates cardiac function (McClellan et al., 2001; Harris et al., 2002; Dhandapany et al., 2009; Girolami et al., 2010). MyBP-C dysfunction is associated with HCM (Harris et al., 2002; Girolami et al., 2010). In embryonic zebrafish, knockdown of *mybpc3* with morpholinos leads to cardiac hypertrophy, diastolic HF and pericardial edema (Chen et al., 2013). Cardiac hyperplasia was not reported.

As the above examples demonstrate, dysfunction of the sarcomere often leads to HF. In the embryonic lethal zebrafish HF mutant *main squeeze (msq)* stress-responsive genes are severely down-regulated (Bendig et al., 2006). HF in these mutants is due to a mutation in *integrin-linked kinase (ilk)*, which reduces ILK kinase activity and disrupts binding to a sarcomeric adaptor protein. Cardiac contractility quantified as fractional shortening is rescued by multiple players in this pathway. This study elegantly shows how hearts sense mechanical stretch and respond to it (Bendig et al., 2006).

Noonan syndrome (NS) and LEOPARD (LS) syndrome are developmental disorders caused by autosomal dominant mutations in *PTPN11*, which encodes Shp2, a ubiquitously expressed non-receptor protein-tyrosine phosphatase that is involved in numerous signal transduction pathways. NS and LS are characterized by congenital heart defects and HCM. To investigate the pathomechanisms of these developmental disorders, NS and LS *Hsp2*-variants were expressed in embryonic zebrafish, which exhibit elongated heart tubes, reduced cardiomyocyte migration and impaired leftward displacement of the heart (Bonetti et al., 2014). Ciliogenesis and cilia function in Kupffer’s vesicle is impaired, as a result of hyperactivation of MAPK signaling. Inhibition of MAPK signaling prior to gastrulation rescues cilia length and heart laterality defects. This study shows how the pathomechanisms of developmental disorders may be investigated with embryonic zebrafish. Some autosomal-recessive NS patients show variations in Leucine-zipper-like transcription regulator 1 (LZTR1). CRISPR-Cas9 genome editing was used to generate *lztr1*-mutant zebrafish (Nakagama et al., 2020). These adult zebrafish phenocopy the human disease, showing ventricular hypertrophy and vascular malformations, demonstrating that also adult zebrafish models are relevant for investigating congenital heart disease.

*PKD2* encodes polycystin-2 (PC2), a non-selective calcium-regulated cation channel expressed on the endoplasmic/sarcoplasmic membrane and in primary cilia. In cardiomyocytes it participates in intracellular calcium handling. Mutations in *PKD2* cause autosomal dominant polycystic kidney disease (ADPKD) (Mochizuki et al., 1996). Knockdown of *pkd2* with morpholinos in embryonic zebrafish results in reduced cardiac contractile function and atrioventricular block due to impaired cardiomyocyte calcium cycling (Paavola et al., 2013). *PKD2* mutations are associated with DCM, and this study gives insights into possible pathomechanisms of HF associated with ADPKD.

Septins are small GTPases associated with actin filaments and play an important role in cytoskeleton organization. Knockdown of *sept7b* with morpholinos in embryonic zebrafish reveals reduced expression of actin and sarcomeric disassembly leading to reduced ventricular size and contractility (Dash et al., 2017).

Vascular endothelial zinc finger 1 (*vezf1*) is a transcription factor regulating vasculogenesis and angiogenesis. Expression of *vezf1* is decreased in diseased human and murine myocardium (Paavola et al., 2020). Knockdown of *vezf1* with morpholinos in zebrafish embryos impairs cardiac growth and contractile response to beta-adrenergic stimuli suggesting a role for *vezf1* in cardiac remodeling (Paavola et al., 2020).

Screening for novel HF-associated genetic variants revealed a potentially damaging variant of tripartite motif containing 55 (*TRIM55*), encoding an E140K variant. *TRIM55* is a protein involved in sarcomere assembly and is highly expressed in heart and skeletal muscle. Deletion of *trim55a* or overexpression of *trim55* E140K with CRISPR-Cas9 reduces cardiac contractility in embryonic zebrafish (Heliste et al., 2020). This study demonstrates the usability of zebrafish as a principal animal model for investigating candidate genes and genetic variants.

GTP-binding protein 3 (GTPBP3) is an RNA modifying enzyme and mutations in *GTPBP3* are associated with HCM (Kopajtich et al., 2014). *gtpbp3* knockout embryonic zebrafish, generated with CRISPR-Cas9, exhibit impaired mitochondrial translation leading to cardiac hypertrophy, impaired contractility, and myocardial disarray (Chen et al., 2019). These findings recapitulate the clinical phenotype of HCM patients carrying mutations in *GTPBP3* (Kopajtich et al., 2014).

Leucine-rich repeat containing protein 10 (Lrrc10) is believed to provide structural framework for protein-protein interactions that are essential during cardiac development. Knockdown of *lrrc10* with morpholinos in embryonic zebrafish reveals developmental defects including cardiac looping failure, reduced contractility, pericardial edema and embryonic lethality (Kim et al., 2007). Morphants have less cardiomyocytes, lower ejection fraction, and higher expression levels of atrial natriuretic factor (ANF), whose expression levels are upregulated in cardiac hypertrophy (Rockman et al., 1994).

RNA binding protein, fox-1 homolog (RBFox1), an RNA splicing regulator, is required for postnatal cardiac maturation and is expressed during cardiac development. Knockdown of *rbfox1* in embryonic zebrafish with morpholinos decreases ejection fraction and increases pericardial edema (Gao et al., 2016). RBFox1 is required for splicing of transcription factor myocyte enhancer factor-2 (Mef2) family members yielding Mef2 isoforms with varying effects on cardiac hypertrophic gene expression. This study shows that regulation of RNA splicing by RBFox1 is an important player in transcriptome reprogramming during HF.

Neural crest cells migrate to embryonic hearts and transform into cardiomyocytes (Li et al., 2003; Sato and Yost, 2003). In zebrafish ventricles, neural crest-derived cardiomyocytes (NC-Cms) express a notch ligand, jagged canonical Notch ligand 2b (*jag2b*). Genetic ablation of NC-Cms in embryonic zebrafish reveals altered notch signaling, reduced *jag2b* expression and changes in trabeculation patterns (Abdul-Wajid et al., 2018). The transgenic NC-Cm-depleted zebrafish survive to adulthood. However, as adults these zebrafish show severe HCM and HF upon exercise stress testing. Adult *jag2b* mutant zebrafish show similar cardiac phenotype. These are interesting models for investigating adult-onset HCM and stress-induced HF.

Erb-B2 Receptor Tyrosine Kinase 2 (Erbb2) plays an important role in trabeculae formation (Lee et al., 1995; Liu et al., 2010). Trabeculae-deficient *erbb2* mutant embryonic zebrafish develop HCM-like phenotype for compensation of aberrant trabeculae formation (Fleming et al., 2018). Rapamycin treatment rescues impaired cardiac contractility and cardiac hypertrophy through the inhibition of target of rapamycin (TOR) pathway. In line with these results, previously a transgenic zebrafish line with dominant negative expression of *erbb2* showed DCM-like phenotype and aberrant trabeculae formation (Reischauer et al., 2014). These models enable investigation of the role of trabeculae in cardiac structure and function.

BAG Cochaperone 3 (Bag3) is classified as a cochaperone of the heat shock proteins, thought to assist mainly in degradation of cellular proteins. Mutations in *Bag3* cause DCM in humans. Knockdown of *bag3* by morpholinos in embryonic zebrafish results in impaired contractility and pericardial edema (Norton et al., 2011). In adult zebrafish, knockout of *bag3* with TALEN genome editing technology results in enlarged cardiac chambers, reduced ejection fraction, and activation of mechanistic target of rapamycin (mTOR). Inhibition of mTOR signaling improves cardiac function and hence, could be a target gene for therapeutic treatment of DCM caused by mutations in *Bag3* (Ding et al., 2019). However, CRISPR-Cas9 generated knockout of *bag3* fails to show a DCM phenotype in adult zebrafish due to compensation by *bag2* (Diofano et al., 2020). These studies also demonstrate how genetic compensation may influence the penetrance of disease-causing mutations *in vivo*, and underscores the importance of comparing various gene-editing techniques and solving possible discrepancies.

Lysosome-associated membrane protein 2 (Lamp2) is a membrane protein localized in lysosomes and endosomes (Ding et al., 2020). Mutations in *Lamp2* cause Danon disease in humans, characterized by HCM and accelerated autophagy in tissues. The TALEN-generated *lamp2* knockout adult zebrafish exhibit cardiac hypertrophy, reduced ventricular ejection fraction, increased autophagy, reduced physical exercise capacity, and attenuated β-adrenergic contractile response (Dvornikov et al., 2019). Cardiac hypertrophy and impaired contractile function are partially rescued by inhibition of *mtor*. This study demonstrates the feasibility of modeling inherited HCM in the adult zebrafish.

NADH:Ubiquinone Oxidoreductase Subunit A7 (*NDUFA7*) encodes a subunit of NADH:ubiquinone oxidoreductase (complex I) in the mitochondrial respiratory chain. Hedgehog acyltransferase-like (HHATL), a sarcoplasmic reticulum resident protein, is highly expressed in the heart and thought to be essential for postnatal muscle maturation (Van et al., 2015). Whole exome sequencing of HCM patients found an association with *NDUFA7* and *HHATL* (Xu et al., 2015). In embryonic zebrafish, knockdown of *ndufa7* and *hhatla* (*HHATL* ortholog in zebrafish) with morpholinos results in impaired contractility, cardiac hypertrophy, and increased expression of the hypertrophy markers *nppa* (ANP), *nppb* (BNP), and *vmhc* (ventricular myosin heavy chain) (Shi et al.,2020a,b). In both cases, calcineurin signaling is mechanistically implicated.

The heart development protein with EGF-like domains 1 (HEG1) receptor is expressed in endothelial cells and endocardium cells (Kleaveland et al., 2009). HEG1 is an important intercellular adhesion cadherin protein, maintaining cardiovascular function during embryonic development. Embryonic zebrafish *heg1* mutants show signs of severe HF (Mably et al., 2003). *heg1* knockout zebrafish generated with CRISPR-Cas9 show atrial and ventricular enlargement, slow heart rate and blood flow, and pericardial edema (Lu et al., 2020). The authors used this DCM model for drug screening. However, no data on cardiac contractility is reported.

Skeletal muscle growth 5 (*USMG5*) encodes Diabetes-associated protein in insulin-sensitive tissue (DAPIT). DAPIT is a component of ATP synthase, playing an important role in energy production. Knockdown of *z-usmg5* with morpholinos in embryonic zebrafish results in impaired cardiac contractility, atrial enlargement, and pericardial edema (Nagata et al., 2017). This study suggests that insufficient energy production may lead to a DCM-like phenotype and HF.

Zebrafish mutant *tr265*/*tr265*, identified from an ENU mutagenesis screen, has malformed erythrocytes due to a solute carrier family 4 member 1 (*slc4a1a*) mutation leading to anemia and high-output cardiac stress (Sun et al., 2009). Hearts of the *tr265/tr265* mutants show hypertrophy at 4 weeks post-fertilization and hyperplasia later at 16 weeks post-fertilization. This study is the first to show that unlike in mammalian models, both cardiomyocyte hypertrophy and hyperplasia contribute to the cardiac remodeling process in zebrafish. Adult zebrafish carrying mutations in atrial myosin heavy chain (*myh6^*s*459^*) have a weak atrium-phenotype. The atrium remains hypoplastic and shows elastin deposition while mutant ventricles exhibit increased size due to cardiomyocyte hyperplasia (Sarantis et al., 2019). Cardiac growth and vascularization are interlinked during development. Overexpression of the angiogenic factor *vegfaa* induces cardiac growth with hyperplasia in adult zebrafish (Karra et al., 2018). Gene-trap identified vinculin b (*vclb*) mutants, that were additionally generated with CRISPR-Cas9 technology, display multiple cardiac defects and abnormal coronary vessel development (Cheng et al., 2016). Epicardium-derived cells including cardiomyocytes overproliferate in *vclb* mutant fish. These, as well as some of the above-mentioned studies indicate that zebrafish respond to cardiac stress/injury with cardiomyocyte hyperplasia in addition to hypertrophy.

Mitochondrial calcium uniporter (MCU) is the major route for calcium uptake into the mitochondrial matrix (Baughman et al., 2011; De Stefani et al., 2011). Mitochondrial calcium uptake is essential for energy production, cell survival, and is shown to regulate cardiac calcium signaling and physiology (Drago et al., 2012; Wu et al., 2015). Adult zebrafish *mcu*^la2446^ mutants, generated with TALEN technology, exhibit weakly contracting hypoplastic ventricles, sinus arrest, defects in the conduction system, as well as swollen mitochondria and damaged myofibrils in the ventricles (Langenbacher et al., 2020). This model is relevant for investigating mitochondrial calcium handling in the heart. Atrial fibrillation (AF) is the most common clinical arrhythmia; however, the underlying mechanisms of AF remain incompletely understood. Transgenic *cmlc1* (*cmlc1 is the* zebrafish ortholog of atrial-specific myosin light chain, *MYL4*) mutant adult zebrafish hearts display disrupted sarcomeric structure, atrial enlargement, and electrical abnormalities associated with human AF (Orr et al., 2016). These findings describe the cause of a rare subtype of AF due to a primary, atrial-specific sarcomeric defect. *dco^*s*226^* embryonic zebrafish mutants, identified from an ENU mutagenesis screen, develop HF due to interrupted morphogenesis following uncoordinated ventricular contraction, as investigated with optical mapping/calcium imaging (Chi et al., 2010). *dco* encodes the gap junction protein Gja3/Cx46. Mouse *Cx46* mutants show cardiac conduction defects associated with human HF (Chi et al., 2010). This interesting study shows that cardiac electrical forces are required to preserve cardiac chamber morphology and may act as an important epigenetic factor in cardiac remodeling. Together these studies demonstrate the usability of zebrafish to model human arrhythmias and conduction defects.

Non-syndromic mitral valve prolapse (MVP) is a common cardiac valvular disease manifested by mitral regurgitation and HF (Guy and Hill, 2012). Genome-wide association studies have identified candidate genes for MVP (Dina et al., 2015). These include LIM and cysteine-rich domains 1 (*LMCD1*), a co-regulator of transcription highly expressed in cardiac tissue and a direct regulator of *Gata6* in mice (Rath et al., 2005). Knockdown of *lmcd1* with morpholinos in embryonic zebrafish results in significantly increased atrioventricular regurgitation (Dina et al., 2015). Another candidate gene, tensin 1 (*TNS1*), encodes a focal adhesion protein involved in cytoskeleton organization. *TNS1* variants have been linked to a rare X-linked form of MVP (Kyndt et al., 2007). Knockdown of *tns1* with morpholinos in embryonic zebrafish results in a similar phenotype of atrioventricular regurgitation (Dina et al., 2015). This study demonstrates the feasibility of modeling human valvular heart defects in zebrafish.

## Drug-Induced Heart Failure Models

β-adrenergic receptor (β-AR) signaling is known to be dysregulated in human HF. In rodents, acute or chronic β-AR activation by isoproterenol (ISO) causes myocardial damage leading to cardiac dysfunction and remodeling, hence it represents an established HF model (Grimm et al., 1998; Heather et al., 2009; Wang et al., 2019). In adult zebrafish, chronic ISO-treatment for 14 days induces severe systolic cardiac dysfunction similar to mammals, accompanied by transcriptional changes of β-AR components, increased gene expression of *ANP* and *BNP*, increased cell death, elevated inflammation and impaired calcium handling (Kossack et al., 2017). However, as a difference to mammalian models, no fibrosis is detected. In embryonic zebrafish, chronic ISO-treatment for 5 days induces systolic cardiac dysfunction and gene expression of *ANP* and *BNP* (Kossack et al., 2017). Thus, ISO stimulation in embryonic and adult zebrafish is feasible for studying the pathophysiological mechanisms underlying cardiac hypertrophy, cardiac remodeling and HF.

Phenylephrine (PE) is selective α_1_-adrenergic receptor activator. Treatment with PE increases blood pressure and causes cardiac hypertrophy in mice by increasing ventricular afterload (Iaccarino et al., 2001). PE treatment induces hypertrophy in *ex vivo* cultured adult zebrafish hearts (Romano and Ceci, 2020). Treatment of *ex vivo* zebrafish hearts with PE induces cardiomyocyte hypertrophy and epicardial hyperplasia as indicated by phalloidin staining of actin filaments and Wilms’ tumor suppressor (WT1, embryonic epicardial marker) staining of the epicardium (Romano and Ceci, 2020). This *ex vivo* zebrafish cardiac hypertrophy model is similar to mammalian models and may offer advantages for studying cardiac hypertrophy due to easy manipulation of experimental conditions.

Aristolochic Acid (AA) is a phytochemical commonly found in the Aristolochiaceae family of flowering plants. AA is a component of Chinese herbs and is known to be toxic to multiple organs (Huang et al., 2007). In embryonic zebrafish, treatment with AA causes cardiac hypertrophy and gradual loss of contractility. This worsening HF is lethal (Huang et al., 2007, 2014). Histological and electron microscopic studies of the heart samples reveal loss of endocardium, hypertrophy of cardiomyocytes and disorganized cardiac fibers (Huang et al., 2007). The authors also show the potential of this model for drug discovery and identify three compounds that attenuate HF; mitogen-activated protein kinase 1 (MEK-1), a chalcone derivative C25, and a phenolic compound A11 (Huang et al., 2013). Another study, using the same AA-induced HF model, shows that treatment with empagliflozin, a sodium-glucose cotransporter 2 (SGLT2) inhibitor, used in the treatment of type 2 diabetes and HF, attenuates cardiac morphological changes, and reduces the expression of BNP and ANP as well as mortality of embryonic zebrafish (Shi et al., 2017).

Exposure to polycyclic aromatic hydrocarbons (PAHs) by pollution associates with cardiac pathologies such as hypertrophy, arrhythmias and contractile dysfunction (Marris et al., 2020). In zebrafish, exposure to PAHs during embryonic development is cardiotoxic and leads to adverse effects on heart development (Hicken et al., 2011; Incardona et al., 2011). A high-ring PAH, benzo[a]pyrene (BaP) causes bradycardia and pericardial edema at high concentrations (Incardona et al., 2011). Exposure to low dose BaP during embryonic development causes cardiac hypertrophy in adult zebrafish as revealed by increased heart weight to body weight ratio, deposition of collagen in the heart, and elevated gene expression of ANP, BNP, and proto-oncogene c-Myc (Huang et al., 2014). Similarly, transient exposure of embryonic zebrafish to low concentrations of crude oil affects heart function at later stages (Hicken et al., 2011).

Phenylhydrazine hydrochloride (PHZ) is a small molecule that induces anemia through lysis of red blood cells (Norman and Mc, 1958). Chronic anemia leads to cardiomegaly resulting from both hypertrophy and hyperplasia in adult zebrafish (Sun et al., 2009). Fractional shortening is reduced and ventricular diameter is increased after 5 weeks of PHZ treatment (Ernens et al., 2018). However, as zebrafish has tremendous capability to recover from HF, cardiac function is restored to baseline levels 3 weeks after PHZ treatment withdrawal. Hence, this model may be used to investigate mechanisms of cardiac repair.

Verapamil is a calcium channel blocker that is used for treating cardiac arrhythmias, hypertension, and angina. Overdose of verapamil causes HF in humans. Zhu et al. (2018) developed a verapamil-induced embryonic zebrafish HF model for drug screening. Treatment with verapamil causes pericardial edema and venous blood congestion with reduced cardiac output and blood flow velocity (Zhu et al., 2018). The model was validated by testing the efficacy of 8 human HF drugs, which all show significant therapeutic effect on the zebrafish HF model. This embryonic zebrafish HF model may thus be used for *in vivo* drug screening.

Tolterodine, a muscarinic receptor antagonist, has been identified as a modifier of cardiac conduction in embryonic zebrafish (Burczyk et al., 2019). Treatment with tolterodine leads to decreased heart rate, pericardial edema and arrhythmia. Additionally, it induces expression of Tbx18, which is essential for differentiation of contractile cardiomyocytes into pacemaker cells. Targeted inhibition of muscarinic receptors with tolterodine may induce new pacemaker cells in the adult heart and ameliorate cardiac arrhythmias (Burczyk et al., 2019). Thus, this model is useful for investigating the cardiac conduction system.

Terfenadine is a commonly used antihistamine for treating allergies. However, treatment with terfenadine involves a risk of developing arrhythmias. Gu et al. (2017) developed a terfenadine-induced embryonic zebrafish DCM model. Terfenadine treated zebrafish show reduced circulation, heart rate and cardiac contractility, atrioventricular block, pericardial edema and enlarged ventricular area (Gu et al., 2017). Additionally, cardiomyocyte apoptosis and gene expression of BNP are increased. This rapid and simple model may be used for drug screening and toxicity assays for non-ischemic HF.

Anthracyclines are commonly used anticancer drugs with serious side effects including cardiotoxicity. Developmental cardiotoxicity of anthracyclines has been investigated in embryonic zebrafish (Han et al., 2015), which show similar dose-dependent effects on the heart as mammalian models. Doxorubicin is a highly effective anthracycline class chemotherapy agent. A doxorubicin-induced CM model in embryonic zebrafish recapitulates the cardiomyocyte apoptosis and decline in contractility seen in human patients (Liu et al., 2014). This model may be used for screening cardioprotective drugs and investigating cardioprotective mechanisms.

## Other Heart Failure Models

As HF commonly develops following cardiac injury, namely myocardial infarction (MI), various models have been developed in zebrafish to model MI. The first model was the ground-breaking observation that zebrafish regenerates its heart after resection of 20% of the ventricle (Poss et al., 2002). After that the cryoinjury model, in which the dead and injured cells remain in the injury area, was developed (Chablais et al., 2011; Gonzalez-Rosa et al., 2011; Schnabel et al., 2011). The third common method to model MI is genetic ablation of cardiomyocytes driven either by nitroreductase (Curado et al., 2007) or diphtheria toxin (Wang et al., 2011). Additionally, ischemic cardiac injury in adult zebrafish has been induced by hypoxia/reoxygenation (Parente et al., 2013). 15 min in hypoxic conditions results in oxidative stress, inflammation, death and proliferation of cardiomyocytes. Fractional area change decrease measured 1 day post-injury, is fully recovered a month post-injury. These models have been used mainly to focus on the mechanisms of the regenerative capability of the zebrafish heart. This regenerative capability limits using the MI models for investigating chronic HF, as the impaired cardiac function recovers in a few weeks post-injury.

Exercise and physical stress are known to cause adaptive cardiac hypertrophy in humans (Nakamura and Sadoshima, 2018). Conversely, in zebrafish exercise-induced cardiomegaly is the result of hyperplasia, not hypertrophy (Jean et al., 2012). Cardiac function prevails during this adaptive cardiac hyperplasia. Thus, while unable to model HF, this model is relevant for investigating cardiac remodeling. However, others report zebrafish exercise-induced cardiomegaly to result from hypertrophy, oblivious to the possibility of cardiac hyperplasia (Zhou et al., 2020). The authors also describe notable cardiac contractile impairment in the exercised fish. However, no data on this is presented (Zhou et al., 2020). The effects of exercise have been studied in a zebrafish cardiac cryoinjury model (Rovira et al., 2018). Exercise improves cardiac function and scar tissue clearance post-injury, and increases cardiomyocyte proliferation. No cardiac damage or hypertrophy is reported. Additionally, exercise-induced physical stress can be used to bring out the pathological phenotype in those with a genetic predisposition. At baseline phenotypically unremarkable heterozygous zebrafish adults carrying mutations associated with CM in myosin light chain develop HF in response to physical stress (Scheid et al., 2016). This study demonstrates the usefulness of exercise-induced physical stress for revealing pathophysiology when cardiac function remains compensated under resting conditions.

Hyperglycemia predisposes to HF (Bell, 2003). Many zebrafish diabetes models have been published (Zang et al., 2018). In embryonic zebrafish, knockdown of *glut12* with morpholinos causes hyperglycemia and HF (Jimenez-Amilburu et al., 2015). However, in this model of diabetic CM, abnormal valve formation and bradycardia are concluded to indicate HF, without showing data on cardiac size or pump function. A hyperglycemia-induced model of diabetic CM in adult zebrafish induces cardiac hypertrophy and impaired diastolic function followed by impaired systolic function (Sun et al., 2017). In another diabetic CM model, hyperglycemia is induced by intraperitoneal streptozocin injections, leading to heart enlargement, arrhythmias, and diastolic dysfunction followed by systolic dysfunction (Wang et al., 2020). These models are relevant for investigating diabetic CM and HF. Additionally, the effects of hyperglycemia on congenital heart defects have been investigated in developing zebrafish embryos (Liang et al., 2010).

## Discussion

Heart failure is characterized either by impaired pump/systolic function of the heart [heart failure with reduced ejection fraction (HFrEF)] or impaired relaxation/diastolic function of the heart [heart failure with preserved ejection fraction (HFpEF)] (Ponikowski et al., 2016). HFpEF is becoming the predominant form of HF, and these patients are more likely to be women of advanced age with comorbidities (Dunlay et al., 2017). The etiology of HFpEF remains incompletely understood, and prognosis-improving drugs are lacking. When modeling HF, it is therefore important to characterize its sub-type as accurately as possible. Up until recently, zebrafish HF models have largely remained uncharacterized with regard to diastolic and/or systolic dysfunction. However, characterization of the HF sub-type in adult zebrafish is becoming more common with the development and availability of echocardiography (Lee et al., 2014; Ernens et al., 2016; Wang et al., 2017). For adult zebrafish echocardiography, the time has come to establish guidelines for standardized imaging conditions (anesthesia, water temperature etc.) and criteria for characterizing cardiac function. Additionally, embryonic zebrafish HF models now often elaborate on the sub-type of HF (Chen et al., 2013).

With chronic stress, HF develops slowly during a process of cardiac remodeling. Human adult cardiomyocytes are terminally differentiated, and cannot increase in number in response to stress. Instead, the cardiomyocytes increase in size (cardiac hypertrophy) to maintain sufficient heart function to meet the body’s demands. With time, this adaptive hypertrophy becomes maladaptive and progresses to HF via numerous molecular signaling pathways, which still remain largely elusive (McMullen and Jennings, 2007; Tham et al., 2015). These pathways leading to cardiac remodeling are known to involve modulation of cell growth and proliferation, gene expression (also by non-coding RNAs), immune responses, cellular metabolism, mitochondrial function, fibrosis, impaired intracellular calcium handling (Lou et al., 2012), cell death etc. The question of whether cardiac hypertrophy is beneficial (adaptive) or harmful (maladaptive) continues to trouble scientists (Carabello, 2014). Unlike humans, zebrafish hearts are able to respond to cardiac stress by proliferation of cardiomyocytes (hyperplasia) in addition to growth of cell size (hypertrophy), as shown for high-output cardiac stress due to anemia (Sun et al., 2009) and following ventricular resection (Karra et al., 2018). Quantification of cardiomyocyte numbers and sizes in embryonic zebrafish hearts is relatively straight-forward (Figure 2). Additionally, for quantifying cardiomyocyte number, transgenic zebrafish such as Tg (myl7:DsRed2-nuc) are useful (Miura and Yelon, 2011). This makes zebrafish an intriguing model for investigating the detailed mechanisms of adaptive versus maladaptive cardiac remodeling. However, understanding the similarities and differences of these remodeling pathways in human and in zebrafish is essential.

Zebrafish is a useful model for *in vivo* high-throughput drug and toxicity screening, and has been used for cardiac drug discovery (Kessler et al., 2015), investigation of drug-induced cardiotoxicity (Zakaria et al., 2018), as well as nanoparticle toxicity (Chakraborty et al., 2016). Importantly, drug-induced effects on the QT-interval show excellent correlation between human and zebrafish (Milan et al., 2006). Drugs used for treatment of HF have been shown to be beneficial for preventing acute HF in an embryonic zebrafish HF model (Zhu et al., 2018). Thus, from a pharmacological point of view, zebrafish offers a relevant vertebrate model for drug-screening and drug-induced HF.

Cardiac electrophysiology, namely heart rate and action potential duration, is qualitatively similar in zebrafish and humans (Arnaout et al., 2007; Nemtsas et al., 2010). The fundamentals of cardiac excitation-contraction coupling, conversion of the electrical signal (action potential) to the mechanical response (contraction), is conserved between humans and zebrafish (Bovo et al., 2013). Combined with the high degree of genetic similarity (Barbazuk et al., 2000), this conserved physiology allows zebrafish to be used for modeling human genetic and acquired CMs (Dahme et al., 2009; Gut et al., 2017; Ding et al., 2020). This supports the use of zebrafish as a first-line animal model for investigating phenotype and physiology of genetic variants, as has been successfully done for human primary genetic CMs: DCM (Shih et al., 2015), HCM (Dvornikov et al., 2018), restrictive cardiomyopathy (RCM) (Louw et al., 2018), arrhythmogenic right ventricular cardiomyopathy (ARVC) (Asimaki et al., 2014), and left ventricular non-compaction cardiomyopathy (LVNC) (Bainbridge et al., 2015).

However, it is essential to acknowledge the caveats of using zebrafish to model human heart disease (Table 1B). When modeling human HF, essential differences in hemodynamics are important to take into account. The zebrafish heart is two-chambered and lacks pulmonary circulation, obviously limiting chances to model human right-sided HF. In fish, ventricular filling during diastole is mainly determined by atrial contraction, as opposed to central venous pressure in humans (Cotter et al., 2008).

Although the fundamentals of cardiac electrophysiology are similar in zebrafish and humans, important differences exist. In zebrafish the balance of inward currents is different; sodium current is lower and calcium current higher than in humans. In repolarization, outward potassium currents differ because the transient outward potassium current and the slow component of the delayed rectifier current are absent in zebrafish (Vornanen and Hassinen, 2016). However, human genetic repolarization disorders, both short and long QT syndromes, have been successfully modeled in zebrafish (Milan et al., 2006; Arnaout et al., 2007; Thorsen et al., 2017). Some have concluded that zebrafish may provide a relevant model for cardiac electrophysiology associated with abnormal repolarization, but may be less suitable for studying depolarization disorders or calcium-modulated arrhythmias (Verkerk and Remme, 2012). Almost half of HF patients suffer sudden cardiac death (SCD) mainly due to ventricular tachyarrhythmias, putting SCD on par with failure of pump function as a cause of mortality in HF (Tomaselli et al., 1994). It follows that a better understanding of zebrafish cardiac electrophysiology and its limitations is needed for relevant modeling of human cardiac electrophysiology and arrhythmias associated with HF.

Impaired calcium handling plays an essential role behind both weakened cardiac pump function and arrhythmias in the failing human heart (Pogwizd and Bers, 2002). Dysregulation of calcium, in addition to triggering arrhythmias by causing afterdepolarizations, is also known to contribute to impaired cardiac function and formation of anatomical substrate for arrhythmias by increased fibrosis (Chen et al., 2005; Nakayama et al., 2007). In human ventricular cardiomyocytes, the majority of calcium responsible for muscle contraction comes from the intracellular stores of the sarcoplasmic reticulum (SR), whereas in zebrafish SR calcium release is limited, accounting for only a fraction of the calcium transient (Bovo et al., 2013). In zebrafish, the main source of calcium for muscle contraction is extracellular calcium, which enters mainly via sarcolemmal T-type calcium channels (Vornanen and Hassinen, 2016; Haverinen et al., 2018). In humans, the sarcolemmal calcium current necessary for calcium-induced calcium release occurs via L-type calcium channels, whereas significant expression of T-type calcium channels is lacking (Gaborit et al., 2007). At the cellular level, adult zebrafish ventricular cardiomyocytes more resemble their neonatal than adult mammalian counterparts. Zebrafish cardiomyocytes are clearly thinner, lack sarcolemmal T-tubules, have lower dependence on SR calcium cycling and larger dependence on sarcolemmal T-type calcium currents for muscle contraction (Haverinen et al., 2018). In healthy mammalian hearts, the force-frequency relationship (FFR) of cardiac muscle contraction is positive, i.e., the force of muscle contraction increases as the heart rate increases. A flat or negative FFR is a hallmark of HF (Pieske et al., 1998). Conversely, FFR is strongly negative in the zebrafish heart (Haustein et al., 2015), likely mainly due to low dependence of excitation-contraction coupling on SR calcium cycling. These differences need to be acknowledged when modeling human HF.

In zebrafish, gene duplication has resulted in gene paralogs that are not present in mammals (Howe et al., 2013). These paralogs may obtain novel or more limited roles, driving evolution and potentially producing genes without mammalian orthologs. Thus, gene duplication confounds the use of zebrafish to model mammalian genetic diseases (Genge et al., 2016). Furthermore, the rapid development or powerful gene-editing techniques necessitates continuous assessment of the advantages and disadvantages of each method. CRISPR-generated knockout of target genes might paradoxically result in milder phenotypes than transient morpholino-generated knockdown of the same target genes, due to transcriptional adaptation-derived compensation, where related genes are upregulated independently of protein feedback loops (El-Brolosy et al., 2019). These findings may help in designing mutant alleles that minimize this genetic compensation. On the other hand, the phenotypes in morpholino-generated knockdowns may be due to off-target or toxic effects of the reagents used knocking down the gene (Schulte-Merker and Stainier, 2014). For distinguishing possible off-target effects of MOs from specific phenotypes, MOs should be used according to established guidelines (Stainier et al., 2017).

Another aspect limiting the usability of zebrafish for modeling human HF is the apparent scarcity of chronic cardiac fibrosis in zebrafish (Gonzalez-Rosa et al., 2011; Sanchez-Iranzo et al., 2018). In addition to proliferation of cardiac fibroblasts and excessive deposition of extracellular matrix, macrophages are implicated in formation of cardiac fibrosis, leading to stiffening of the cardiac muscle (Simoes et al., 2020). In humans, cardiac fibrosis is closely associated with remodeling following cardiac injury such as MI, and is an important factor contributing to development of HF and arrhythmias involving conduction abnormalities (Kashani and Barold, 2005; de Jong et al., 2011; Braunwald, 2013). Fibrosis along with increased heart size delays conduction and contributes to forming an anatomical substrate for reentrant arrhythmias. Although zebrafish lack a specialized His-Purkinje conduction system, they possess ventricular cardiomyocytes expressing cx46, interpreted as the functional equivalent of the mammalian His-Purkinje system (Chi et al., 2010). With small hearts, differences in intracellular calcium cycling, and scarce fibrosis, the anticipated value of modeling structural arrhythmias and chronic cardiac fibrosis related to HF in zebrafish is limited. However, some do report significant chronic cardiac fibrosis in zebrafish (Huang et al., 2014; Sun et al., 2020). In addition to scarcity of chronic cardiac fibrosis in zebrafish, the same applies to lack of cardiac fatty tissue, a hallmark of human arrhythmogenic CM (Gerull and Brodehl, 2020). From another viewpoint, the apparent scarcity of chronic fibrosis and fatty tissue in zebrafish provides an excellent model system for studying the detailed molecular mechanisms of cardiac repair and regeneration, as the increasing research efforts of recent years indicate (Foglia and Poss, 2016; Karra and Poss, 2017; Sanz-Morejon and Mercader, 2020). Therefore, while modeling especially chronic human HF in zebrafish is in many aspects suboptimal, zebrafish provide an excellent model for investigating how to repair and regenerate the diseased heart.

## Conclusion

Based on genotypic and phenotypic similarities, zebrafish provide a relevant model for investigating CMs and the phenotype/physiology of genetic variants. When its limitations are carefully acknowledged, zebrafish can also be useful for modeling pathological cardiac electrophysiology, and congenital heart disease (Gut et al., 2017). With each model, attention should be paid to characterize cardiac structure and function as accurately as possible, including quantification of cardiac size while distinguishing between cardiac hypertrophy and hyperplasia.

Factors limiting the value of zebrafish HF models for impaired cardiac function and arrhythmias include zebrafish having small two-chambered hearts with scarce fibrosis, and differences in calcium cycling and ionic currents, which lead to differences in hemodynamics and cardiac electrophysiology.

The most interesting zebrafish HF research avenue is the tremendous ability of zebrafish to repair and restore cardiac function of the failing heart and thus recover from HF. Diving into the depths of these molecular pathways with zebrafish will hopefully provide significant insights for advancing treatment of human HF in the years to come.

## Author Contributions

SN and HW wrote the initial draft of the manuscript. SN and SP prepared the figures. IT (genetic models) and PL (drug-induced models) wrote the manuscript. JP wrote the rest of the manuscript and revised the final manuscript. All authors have approved submission of the manuscript.

## Conflict of Interest

The authors declare that the research was conducted in the absence of any commercial or financial relationships that could be construed as a potential conflict of interest. The reviewer DB declared a past collaboration with one of the authors PL to the handling editor.

## References

[B1] Abdul-Wajid S. Demarest B. L. Yost H. J. (2018). Loss of embryonic neural crest derived cardiomyocytes causes adult onset hypertrophic cardiomyopathy in zebrafish. *Nat. Commun.* 9:4603. 10.1038/s41467-018-07054-8 30389937 PMC6214924

[B2] Arnaout R. Ferrer T. Huisken J. Spitzer K. Stainier D. Y. Tristani-Firouzi M. (2007). Zebrafish model for human long QT syndrome. *Proc. Natl. Acad. Sci. U.S.A.* 104 11316–11321. 10.1073/pnas.0702724104 17592134 PMC2040896

[B3] Arrenberg A. B. Stainier D. Y. Baier H. Huisken J. (2010). Optogenetic control of cardiac function. *Science* 330 971–974. 10.1126/science.1195929 21071670

[B4] Asimaki A. Kapoor S. Plovie E. Karin Arndt A. Adams E. Liu Z. (2014). Identification of a new modulator of the intercalated disc in a zebrafish model of arrhythmogenic cardiomyopathy. *Sci. Transl. Med.* 6:240ra274. 10.1126/scitranslmed.3008008 24920660 PMC4471875

[B5] Bainbridge M. N. Davis E. E. Choi W. Y. Dickson A. Martinez H. R. Wang M. (2015). Loss of function mutations in NNT are associated with left ventricular noncompaction. *Circ. Cardiovasc. Genet.* 8 544–552. 10.1161/CIRCGENETICS.115.001026 26025024 PMC4545476

[B6] Barbazuk W. B. Korf I. Kadavi C. Heyen J. Tate S. Wun E. (2000). The syntenic relationship of the zebrafish and human genomes. *Genome Res.* 10 1351–1358. 10.1101/gr.144700 10984453 PMC310919

[B7] Baughman J. M. Perocchi F. Girgis H. S. Plovanich M. Belcher-Timme C. A. Sancak Y. (2011). Integrative genomics identifies MCU as an essential component of the mitochondrial calcium uniporter. *Nature* 476 341–345. 10.1038/nature10234 21685886 PMC3486726

[B8] Becker J. R. Deo R. C. Werdich A. A. Panàkovà D. Coy S. MacRae C. A. (2011). Human cardiomyopathy mutations induce myocyte hyperplasia and activate hypertrophic pathways during cardiogenesis in zebrafish. *Dis. Model. Mech.* 4 400–410. 10.1242/dmm.006148 21245263 PMC3097461

[B9] Becker J. R. Robinson T. Y. Sachidanandan C. Kelly A. E. Coy S. Peterson R. T. (2012). In vivo natriuretic peptide reporter assay identifies chemical modifiers of hypertrophic cardiomyopathy signalling. *Cardiovasc. Res.* 93 463–470. 10.1093/cvr/cvr350 22198505 PMC3410427

[B10] Bell D. S. (2003). Heart failure: the frequent, forgotten, and often fatal complication of diabetes. *Diabetes Care* 26 2433–2441. 10.2337/diacare.26.8.2433 12882875

[B11] Bendig G. Grimmler M. Huttner I. G. Wessels G. Dahme T. Just S. (2006). Integrin-linked kinase, a novel component of the cardiac mechanical stretch sensor, controls contractility in the zebrafish heart. *Genes Dev.* 20 2361–2372. 10.1101/gad.1448306 16921028 PMC1560411

[B12] Bleumink G. S. Knetsch A. M. Sturkenboom M. C. Straus S. M. Hofman A. Deckers J. W. (2004). Quantifying the heart failure epidemic: prevalence, incidence rate, lifetime risk and prognosis of heart failure the rotterdam Study. *Eur. Heart J.* 25 1614–1619. 10.1016/j.ehj.2004.06.038 15351160

[B13] Bonetti M. Paardekooper Overman J. Tessadori F. Noel E. Bakkers J. den Hertog J. (2014). Noonan and LEOPARD syndrome Shp2 variants induce heart displacement defects in zebrafish. *Development* 141 1961–1970. 10.1242/dev.106310 24718990

[B14] Bovo E. Dvornikov A. V. Mazurek S. R. de Tombe P. P. Zima A. V. (2013). Mechanisms of Ca(2)+ handling in zebrafish ventricular myocytes. *Pflugers. Arch.* 465 1775–1784. 10.1007/s00424-013-1312-2 23821298 PMC4138713

[B15] Braunwald E. (2013). Heart failure. *JACC Heart Fail.* 1 1–20. 10.1016/j.jchf.2012.10.002 24621794

[B16] Burczyk M. S. Burkhalter M. D. Tena T. C. Grisanti L. A. Kauk M. Matysik S. (2019). Muscarinic receptors promote pacemaker fate at the expense of secondary conduction system tissue in zebrafish. *JCI Insight* 4:e121971. 10.1172/jci.insight.121971 31619590 PMC6824298

[B17] Carabello B. A. (2014). Is cardiac hypertrophy good or bad? The answer, of course, is yes. *JACC Cardiovasc. Imaging* 7 1081–1083. 10.1016/j.jcmg.2014.07.013 25459588

[B18] Chablais F. Veit J. Rainer G. Jazwinska A. (2011). The zebrafish heart regenerates after cryoinjury-induced myocardial infarction. *BMC Dev. Biol.* 11:21. 10.1186/1471-213X-11-21 21473762 PMC3078894

[B19] Chakraborty C. Sharma A. R. Sharma G. Lee S. S. (2016). Zebrafish: a complete animal model to enumerate the nanoparticle toxicity. *J. Nanobiotechnology* 14:65. 10.1186/s12951-016-0217-6 27544212 PMC4992559

[B20] Chen D. Zhang Z. Chen C. Yao S. Yang Q. Li F. (2019). Deletion of Gtpbp3 in zebrafish revealed the hypertrophic cardiomyopathy manifested by aberrant mitochondrial tRNA metabolism. *Nucleic Acids Res.* 47 5341–5355. 10.1093/nar/gkz218 30916346 PMC6547414

[B21] Chen X. Zhang X. Kubo H. Harris D. M. Mills G. D. Moyer J. (2005). Ca2+ influx-induced sarcoplasmic reticulum Ca2+ overload causes mitochondrial-dependent apoptosis in ventricular myocytes. *Circ. Res.* 97 1009–1017. 10.1161/01.RES.0000189270.72915.D1 16210547

[B22] Chen Y. H. Pai C. W. Huang S. W. Chang S. N. Lin L. Y. Chiang F. T. (2013). Inactivation of Myosin binding protein C homolog in zebrafish as a model for human cardiac hypertrophy and diastolic dysfunction. *J. Am. Heart Assoc.* 2:e000231. 10.1161/JAHA.113.000231 24047589 PMC3835223

[B23] Cheng F. Miao L. Wu Q. Gong X. Xiong J. Zhang J. (2016). Vinculin b deficiency causes epicardial hyperplasia and coronary vessel disorganization in zebrafish. *Development* 143 3522–3531. 10.1242/dev.132936 27578788

[B24] Chi N. C. Bussen M. Brand-Arzamendi K. Ding C. Olgin J. E. Shaw R. M. (2010). Cardiac conduction is required to preserve cardiac chamber morphology. *Proc. Natl. Acad. Sci. U.S.A.* 107 14662–14667. 10.1073/pnas.0909432107 20675583 PMC2930423

[B25] Cotter P. A. Han A. J. Everson J. J. Rodnick K. J. (2008). Cardiac hemodynamics of the rainbow trout (Oncorhynchus mykiss) using simultaneous Doppler echocardiography and electrocardiography. *J. Exp. Zool. A Ecol. Genet. Physiol.* 309 243–254. 10.1002/jez.453 18366108

[B26] Curado S. Anderson R. M. Jungblut B. Mumm J. Schroeter E. Stainier D. Y. (2007). Conditional targeted cell ablation in zebrafish: a new tool for regeneration studies. *Dev. Dyn.* 236 1025–1035. 10.1002/dvdy.21100 17326133

[B27] Dahme T. Katus H. A. Rottbauer W. (2009). Fishing for the genetic basis of cardiovascular disease. *Dis. Model Mech.* 2 18–22. 10.1242/dmm.000687 19132116 PMC2615162

[B28] Dash S. N. Narumanchi S. Paavola J. Perttunen S. Wang H. Lakkisto P. (2017). Sept7b is required for the subcellular organization of cardiomyocytes and cardiac function in zebrafish. *Am. J. Physiol. Heart Circ. Physiol.* 312 H1085–H1095. 10.1152/ajpheart.00394.2016 28341635

[B29] de Jong S. van Veen T. A. van Rijen H. V. de Bakker J. M. (2011). Fibrosis and cardiac arrhythmias. *J. Cardiovasc. Pharmacol.* 57 630–638. 10.1097/FJC.0b013e318207a35f 21150449

[B30] De Stefani D. Raffaello A. Teardo E. Szabo I. Rizzuto R. (2011). A forty-kilodalton protein of the inner membrane is the mitochondrial calcium uniporter. *Nature* 476 336–340. 10.1038/nature10230 21685888 PMC4141877

[B31] Dhandapany P. S. Sadayappan S. Xue Y. Powell G. T. Rani D. S. Nallari P. (2009). A common MYBPC3 (cardiac myosin binding protein C) variant associated with cardiomyopathies in South Asia. *Nat. Genet.* 41 187–191. 10.1038/ng.309 19151713 PMC2697598

[B32] Dina C. Bouatia-Naji N. Tucker N. Delling F. N. Toomer K. Durst R. (2015). Genetic association analyses highlight biological pathways underlying mitral valve prolapse. *Nat. Genet.* 47 1206–1211. 10.1038/ng.3383 26301497 PMC4773907

[B33] Ding Y. Bu H. Xu X. (2020). Modeling inherited cardiomyopathies in adult zebrafish for precision medicine. *Front. Physiol.* 11:599244. 10.3389/fphys.2020.599244 33329049 PMC7717946

[B34] Ding Y. Dvornikov A. V. Ma X. Zhang H. Wang Y. Lowerison M. (2019). Haploinsufficiency of mechanistic target of rapamycin ameliorates bag3 cardiomyopathy in adult zebrafish. *Dis. Model Mech.* 12:dmm040154. 10.1242/dmm.040154 31492659 PMC6826022

[B35] Diofano F. Weinmann K. Schneider I. Thiessen K. D. Rottbauer W. Just S. (2020). Genetic compensation prevents myopathy and heart failure in an in vivo model of Bag3 deficiency. *PLoS Genet.* 16:e1009088. 10.1371/journal.pgen.1009088 33137814 PMC7605898

[B36] Doyon Y. McCammon J. M. Miller J. C. Faraji F. Ngo C. Katibah G. E. (2008). Heritable targeted gene disruption in zebrafish using designed zinc-finger nucleases. *Nat. Biotechnol.* 26 702–708. 10.1038/nbt1409 18500334 PMC2674762

[B37] Drago I. De Stefani D. Rizzuto R. Pozzan T. (2012). Mitochondrial Ca2+ uptake contributes to buffering cytoplasmic Ca2+ peaks in cardiomyocytes. *Proc. Natl. Acad. Sci. U.S.A.* 109 12986–12991. 10.1073/pnas.1210718109 22822213 PMC3420165

[B38] Dunlay S. M. Roger V. L. Redfield M. M. (2017). Epidemiology of heart failure with preserved ejection fraction. *Nat. Rev. Cardiol.* 14 591–602. 10.1038/nrcardio.2017.65 28492288

[B39] Dvornikov A. V. de Tombe P. P. Xu X. (2018). Phenotyping cardiomyopathy in adult zebrafish. *Prog. Biophys. Mol. Biol.* 138 116–125. 10.1016/j.pbiomolbio.2018.05.013 29884423 PMC6269218

[B40] Dvornikov A. V. Wang M. Yang J. Zhu P. Le T. Lin X. (2019). Phenotyping an adult zebrafish lamp2 cardiomyopathy model identifies mTOR inhibition as a candidate therapy. *J. Mol. Cell Cardiol.* 133 199–208. 10.1016/j.yjmcc.2019.06.013 31228518 PMC6705397

[B41] El-Brolosy M. A. Kontarakis Z. Rossi A. Kuenne C. Gunther S. Fukuda N. (2019). Genetic compensation triggered by mutant mRNA degradation. *Nature* 568 193–197. 10.1038/s41586-019-1064-z 30944477 PMC6707827

[B42] Ernens I. Lumley A. I. Devaux Y. (2018). Restoration of cardiac function after anaemia-induced heart failure in zebrafish. *J. Mol. Cell Cardiol.* 121 223–232. 10.1016/j.yjmcc.2018.07.128 30009777

[B43] Ernens I. Lumley A. I. Devaux Y. Wagner D. R. (2016). Use of coronary ultrasound imaging to evaluate ventricular function in adult zebrafish. *Zebrafish* 13 477–480. 10.1089/zeb.2016.1274 27326768 PMC5124742

[B44] Fink M. Callol-Massot C. Chu A. Ruiz-Lozano P. Izpisua Belmonte J. C. Giles W. (2009). A new method for detection and quantification of heartbeat parameters in Drosophila, zebrafish, and embryonic mouse hearts. *Biotechniques* 46 101–113. 10.2144/000113078 19317655 PMC2855226

[B45] Fleming N. D. Samsa L. A. Hassel D. Qian L. Liu J. (2018). Rapamycin attenuates pathological hypertrophy caused by an absence of trabecular formation. *Sci. Rep.* 8:8584. 10.1038/s41598-018-26843-1 29872120 PMC5988815

[B46] Foglia M. J. Poss K. D. (2016). Building and re-building the heart by cardiomyocyte proliferation. *Development* 143 729–740. 10.1242/dev.132910 26932668 PMC4813344

[B47] Gaborit N. Le Bouter S. Szuts V. Varro A. Escande D. Nattel S. (2007). Regional and tissue specific transcript signatures of ion channel genes in the non-diseased human heart. *J. Physiol.* 582(Pt 2), 675–693. 10.1113/jphysiol.2006.126714 17478540 PMC2075332

[B48] Gao C. Ren S. Lee J. H. Qiu J. Chapski D. J. Rau C. D. (2016). RBFox1-mediated RNA splicing regulates cardiac hypertrophy and heart failure. *J. Clin. Invest.* 126 195–206. 10.1172/JCI84015 26619120 PMC4701548

[B49] GBD 2016 Causes of Death Collaborators (2017). Global, regional, and national age-sex specific mortality for 264 causes of death, 1980-2016: a systematic analysis for the Global Burden of Disease Study 2016. *Lancet* 390 1151–1210. 10.1016/S0140-6736(17)32152-9 28919116 PMC5605883

[B50] Genge C. E. Lin E. Lee L. Sheng X. Rayani K. Gunawan M. (2016). The zebrafish heart as a model of mammalian cardiac function. *Rev. Physiol. Biochem. Pharmacol.* 171 99–136. 10.1007/112_2016_5 27538987

[B51] Gerull B. Brodehl A. (2020). Genetic animal models for arrhythmogenic cardiomyopathy. *Front. Physiol.* 11:624. 10.3389/fphys.2020.00624 32670084 PMC7327121

[B52] Gigli M. Begay R. L. Morea G. Graw S. L. Sinagra G. Taylor M. R. (2016). A review of the giant protein titin in clinical molecular diagnostics of cardiomyopathies. *Front. Cardiovasc. Med.* 3:21. 10.3389/fcvm.2016.00021 27493940 PMC4954824

[B53] Girolami F. Ho C. Y. Semsarian C. Baldi M. Will M. L. Baldini K. (2010). Clinical features and outcome of hypertrophic cardiomyopathy associated with triple sarcomere protein gene mutations. *J. Am. Coll. Cardiol.* 55 1444–1453. 10.1016/j.jacc.2009.11.062 20359594

[B54] Gonzalez-Rosa J. M. Martin V. Peralta M. Torres M. Mercader N. (2011). Extensive scar formation and regression during heart regeneration after cryoinjury in zebrafish. *Development* 138 1663–1674. 10.1242/dev.060897 21429987

[B55] Grimm D. Elsner D. Schunkert H. Pfeifer M. Griese D. Bruckschlegel G. (1998). Development of heart failure following isoproterenol administration in the rat: role of the renin-angiotensin system. *Cardiovasc. Res.* 37 91–100. 10.1016/s0008-6363(97)00212-5 9539862

[B56] Gu G. Na Y. Chung H. Seok S. H. Lee H. Y. (2017). Zebrafish larvae model of dilated cardiomyopathy induced by terfenadine. *Korean Circ. J.* 47 960–969. 10.4070/kcj.2017.0080 29035434 PMC5711688

[B57] Gut P. Reischauer S. Stainier D. Y. R. Arnaout R. (2017). Little fish, big data: zebrafish as a model for cardiovascular and metabolic disease. *Physiol. Rev.* 97 889–938. 10.1152/physrev.00038.2016 28468832 PMC5817164

[B58] Guy T. S. Hill A. C. (2012). Mitral valve prolapse. *Annu. Rev. Med.* 63 277–292. 10.1146/annurev-med-022811-091602 22248324

[B59] Han Y. Zhang J. P. Qian J. Q. Hu C. Q. (2015). Cardiotoxicity evaluation of anthracyclines in zebrafish (*Danio rerio*). *J. Appl. Toxicol.* 35 241–252. 10.1002/jat.3007 24853142

[B60] Harris S. P. Bartley C. R. Hacker T. A. McDonald K. S. Douglas P. S. Greaser M. L. (2002). Hypertrophic cardiomyopathy in cardiac myosin binding protein-C knockout mice. *Circ. Res.* 90 594–601. 10.1161/01.res.0000012222.70819.64 11909824

[B61] Haustein M. Hannes T. Trieschmann J. Verhaegh R. Koster A. Hescheler J. (2015). Excitation-contraction coupling in zebrafish ventricular myocardium is regulated by trans-sarcolemmal Ca2+ influx and sarcoplasmic reticulum Ca2+ release. *PLoS One* 10:e0125654. 10.1371/journal.pone.0125654 25938412 PMC4418605

[B62] Haverinen J. Hassinen M. Dash S. N. Vornanen M. (2018). Expression of calcium channel transcripts in the zebrafish heart: dominance of T-type channels. *J. Exp. Biol.* 221(Pt 10), jeb179226. 10.1242/jeb.179226 29739832

[B63] Heather L. C. Catchpole A. F. Stuckey D. J. Cole M. A. Carr C. A. Clarke K. (2009). Isoproterenol induces in vivo functional and metabolic abnormalities: similar to those found in the infarcted rat heart. *J. Physiol. Pharmacol.* 60 31–39. 19826179

[B64] Heliste J. Chheda H. Paatero I. Salminen T. A. Akimov Y. Paavola J. (2020). Genetic and functional implications of an exonic TRIM55 variant in heart failure. *J. Mol. Cell Cardiol.* 138 222–233. 10.1016/j.yjmcc.2019.12.008 31866377

[B65] Hicken C. E. Linbo T. L. Baldwin D. H. Willis M. L. Myers M. S. Holland L. (2011). Sublethal exposure to crude oil during embryonic development alters cardiac morphology and reduces aerobic capacity in adult fish. *Proc. Natl. Acad. Sci. U.S.A.* 108 7086–7090. 10.1073/pnas.1019031108 21482755 PMC3084145

[B66] Howe D. G. Bradford Y. M. Conlin T. Eagle A. E. Fashena D. Frazer K. (2013). ZFIN, the zebrafish model organism database: increased support for mutants and transgenics. *Nucleic Acids Res.* 41 D854–D860. 10.1093/nar/gks938 23074187 PMC3531097

[B67] Huang C. C. Chen P. C. Huang C. W. Yu J. (2007). Aristolochic Acid induces heart failure in zebrafish embryos that is mediated by inflammation. *Toxicol. Sci.* 100 486–494. 10.1093/toxsci/kfm235 17823451

[B68] Huang C. C. Monte A. Cook J. M. Kabir M. S. Peterson K. P. (2013). Zebrafish heart failure models for the evaluation of chemical probes and drugs. *Assay Drug Dev. Technol.* 11 561–572. 10.1089/adt.2013.548 24351044 PMC3870487

[B69] Huang L. Gao D. Zhang Y. Wang C. Zuo Z. (2014). Exposure to low dose benzo[a]pyrene during early life stages causes symptoms similar to cardiac hypertrophy in adult zebrafish. *J. Hazard. Mater.* 276 377–382. 10.1016/j.jhazmat.2014.05.057 24922095

[B70] Huang P. Xiao A. Zhou M. Zhu Z. Lin S. Zhang B. (2011). Heritable gene targeting in zebrafish using customized TALENs. *Nat. Biotechnol.* 29 699–700. 10.1038/nbt.1939 21822242

[B71] Hwang W. Y. Fu Y. Reyon D. Maeder M. L. Tsai S. Q. Sander J. D. (2013). Efficient genome editing in zebrafish using a CRISPR-Cas system. *Nat. Biotechnol.* 31 227–229. 10.1038/nbt.2501 23360964 PMC3686313

[B72] Iaccarino G. Keys J. R. Rapacciuolo A. Shotwell K. F. Lefkowitz R. J. Rockman H. A. (2001). Regulation of myocardial betaARK1 expression in catecholamine-induced cardiac hypertrophy in transgenic mice overexpressing alpha1B-adrenergic receptors. *J. Am. Coll. Cardiol.* 38 534–540. 10.1016/s0735-1097(01)01396-1 11499749

[B73] Incardona J. P. Linbo T. L. Scholz N. L. (2011). Cardiac toxicity of 5-ring polycyclic aromatic hydrocarbons is differentially dependent on the aryl hydrocarbon receptor 2 isoform during zebrafish development. *Toxicol. Appl. Pharmacol.* 257 242–249. 10.1016/j.taap.2011.09.010 21964300

[B74] Jean M. J. Deverteuil P. Lopez N. H. Tapia J. D. Schoffstall B. (2012). Adult zebrafish hearts efficiently compensate for excessive forced overload cardiac stress with hyperplastic cardiomegaly. *Biores. Open Access.* 1 88–91. 10.1089/biores.2012.0201 23515072 PMC3559224

[B75] Jimenez-Amilburu V. Jong-Raadsen S. Bakkers J. Spaink H. P. Marin-Juez R. (2015). GLUT12 deficiency during early development results in heart failure and a diabetic phenotype in zebrafish. *J. Endocrinol.* 224 1–15. 10.1530/JOE-14-0539 25326603

[B76] Kamisago M. Sharma S. D. DePalma S. R. Solomon S. Sharma P. McDonough B. (2000). Mutations in sarcomere protein genes as a cause of dilated cardiomyopathy. *N. Engl. J. Med.* 343 1688–1696. 10.1056/NEJM200012073432304 11106718

[B77] Karra R. Foglia M. J. Choi W. Y. Belliveau C. DeBenedittis P. Poss K. D. (2018). Vegfaa instructs cardiac muscle hyperplasia in adult zebrafish. *Proc. Natl. Acad. Sci. U.S.A.* 115 8805–8810. 10.1073/pnas.1722594115 30104362 PMC6126768

[B78] Karra R. Poss K. D. (2017). Redirecting cardiac growth mechanisms for therapeutic regeneration. *J. Clin. Invest.* 127 427–436. 10.1172/JCI89786 28145902 PMC5272171

[B79] Kashani A. Barold S. S. (2005). Significance of QRS complex duration in patients with heart failure. *J. Am. Coll. Cardiol.* 46 2183–2192. 10.1016/j.jacc.2005.01.071 16360044

[B80] Kessler M. Rottbauer W. Just S. (2015). Recent progress in the use of zebrafish for novel cardiac drug discovery. *Expert Opin. Drug Discov.* 10 1231–1241. 10.1517/17460441.2015.1078788 26294375

[B81] Kim K. H. Antkiewicz D. S. Yan L. Eliceiri K. W. Heideman W. Peterson R. E. (2007). Lrrc10 is required for early heart development and function in zebrafish. *Dev. Biol.* 308 494–506. 10.1016/j.ydbio.2007.06.005 17601532 PMC2048587

[B82] Kleaveland B. Zheng X. Liu J. J. Blum Y. Tung J. J. Zou Z. (2009). Regulation of cardiovascular development and integrity by the heart of glass-cerebral cavernous malformation protein pathway. *Nat. Med.* 15 169–176. 10.1038/nm.1918 19151727 PMC2665266

[B83] Kopajtich R. Nicholls T. J. Rorbach J. Metodiev M. D. Freisinger P. Mandel H. (2014). Mutations in GTPBP3 cause a mitochondrial translation defect associated with hypertrophic cardiomyopathy, lactic acidosis, and encephalopathy. *Am. J. Hum. Genet.* 95 708–720. 10.1016/j.ajhg.2014.10.017 25434004 PMC4259976

[B84] Kossack M. Hein S. Juergensen L. Siragusa M. Benz A. Katus H. A. (2017). Induction of cardiac dysfunction in developing and adult zebrafish by chronic isoproterenol stimulation. *J. Mol. Cell Cardiol.* 108 95–105. 10.1016/j.yjmcc.2017.05.011 28554511

[B85] Koth J. Maguire M. L. McClymont D. Diffley L. Thornton V. L. Beech J. (2017). High-resolution magnetic resonance imaging of the regenerating adult zebrafish heart. *Sci. Rep.* 7:2917. 10.1038/s41598-017-03050-y 28592901 PMC5462770

[B86] Kyndt F. Gueffet J. P. Probst V. Jaafar P. Legendre A. Le Bouffant F. (2007). Mutations in the gene encoding filamin A as a cause for familial cardiac valvular dystrophy. *Circulation* 115 40–49. 10.1161/CIRCULATIONAHA.106.622621 17190868

[B87] Langenbacher A. D. Shimizu H. Hsu W. Zhao Y. Borges A. Koehler C. (2020). Mitochondrial calcium uniporter deficiency in zebrafish causes cardiomyopathy with arrhythmia. *Front. Physiol.* 11:617492. 10.3389/fphys.2020.617492 33424641 PMC7785991

[B88] Lee J. Cao H. Kang B. J. Jen N. Yu F. Lee C. A. (2014). Hemodynamics and ventricular function in a zebrafish model of injury and repair. *Zebrafish* 11 447–454. 10.1089/zeb.2014.1016 25237983 PMC4172470

[B89] Lee K. F. Simon H. Chen H. Bates B. Hung M. C. Hauser C. (1995). Requirement for neuregulin receptor erbB2 in neural and cardiac development. *Nature* 378 394–398. 10.1038/378394a0 7477377

[B90] Li Y. X. Zdanowicz M. Young L. Kumiski D. Leatherbury L. Kirby M. L. (2003). Cardiac neural crest in zebrafish embryos contributes to myocardial cell lineage and early heart function. *Dev. Dyn.* 226 540–550. 10.1002/dvdy.10264 12619138

[B91] Liang J. Gui Y. Wang W. Gao S. Li J. Song H. (2010). Elevated glucose induces congenital heart defects by altering the expression of tbx5, tbx20, and has2 in developing zebrafish embryos. *Birth Defects Res. A Clin. Mol. Teratol.* 88 480–486. 10.1002/bdra.20654 20306498

[B92] Lin E. Shafaattalab S. Gill J. Al-Zeer B. Craig C. Lamothe M. (2020). Physiological phenotyping of the adult zebrafish heart. *Mar. Genomics* 49:100701. 10.1016/j.margen.2019.100701 31451352

[B93] Liu J. Bressan M. Hassel D. Huisken J. Staudt D. Kikuchi K. (2010). A dual role for ErbB2 signaling in cardiac trabeculation. *Development* 137 3867–3875. 10.1242/dev.053736 20978078 PMC3049280

[B94] Liu Y. Asnani A. Zou L. Bentley V. L. Yu M. Wang Y. (2014). Visnagin protects against doxorubicin-induced cardiomyopathy through modulation of mitochondrial malate dehydrogenase. *Sci. Transl. Med.* 6:266ra170. 10.1126/scitranslmed.3010189 25504881 PMC4360984

[B95] Lou Q. Janardhan A. Efimov I. R. (2012). Remodeling of calcium handling in human heart failure. *Adv. Exp. Med. Biol.* 740 1145–1174. 10.1007/978-94-007-2888-2_52 22453987 PMC3740791

[B96] Louw J. J. Nunes Bastos R. Chen X. Verdood C. Corveleyn A. Jia Y. (2018). Compound heterozygous loss-of-function mutations in KIF20A are associated with a novel lethal congenital cardiomyopathy in two siblings. *PLoS Genet.* 14:e1007138. 10.1371/journal.pgen.1007138 29357359 PMC5794171

[B97] Lu S. Hu M. Wang Z. Liu H. Kou Y. Lyu Z. (2020). Generation and application of the zebrafish heg1 mutant as a cardiovascular disease model. *Biomolecules* 10 1542. 10.3390/biom10111542 33198188 PMC7696531

[B98] Mably J. D. Mohideen M. A. Burns C. G. Chen J. N. Fishman M. C. (2003). heart of glass regulates the concentric growth of the heart in zebrafish. *Curr. Biol.* 13 2138–2147. 10.1016/j.cub.2003.11.055 14680629

[B99] Marris C. R. Kompella S. N. Miller M. R. Incardona J. P. Brette F. Hancox J. C. (2020). Polyaromatic hydrocarbons in pollution: a heart-breaking matter. *J. Physiol.* 598 227–247. 10.1113/JP278885 31840250 PMC7003748

[B100] McClellan G. Kulikovskaya I. Winegrad S. (2001). Changes in cardiac contractility related to calcium-mediated changes in phosphorylation of myosin-binding protein C. *Biophys. J.* 81 1083–1092. 10.1016/S0006-3495(01)75765-7 11463649 PMC1301577

[B101] McKenna W. J. Judge D. P. (2021). Epidemiology of the inherited cardiomyopathies. *Nat. Rev. Cardiol.* 18 22–36. 10.1038/s41569-020-0428-2 32895535

[B102] McMullen J. R. Jennings G. L. (2007). Differences between pathological and physiological cardiac hypertrophy: novel therapeutic strategies to treat heart failure. *Clin. Exp. Pharmacol. Physiol.* 34 255–262. 10.1111/j.1440-1681.2007.04585.x 17324134

[B103] Meng X. Noyes M. B. Zhu L. J. Lawson N. D. Wolfe S. A. (2008). Targeted gene inactivation in zebrafish using engineered zinc-finger nucleases. *Nat. Biotechnol.* 26 695–701. 10.1038/nbt1398 18500337 PMC2502069

[B104] Merrifield G. D. Mullin J. Gallagher L. Tucker C. Jansen M. A. Denvir M. (2017). Rapid and recoverable in vivo magnetic resonance imaging of the adult zebrafish at 7T. *Magn. Reson. Imaging* 37 9–15. 10.1016/j.mri.2016.10.013 27751860 PMC5344283

[B105] Milan D. J. Jones I. L. Ellinor P. T. MacRae C. A. (2006). In vivo recording of adult zebrafish electrocardiogram and assessment of drug-induced QT prolongation. *Am. J. Physiol. Heart Circ. Physiol.* 291 H269–H273. 10.1152/ajpheart.00960.2005 16489111

[B106] Milan D. J. Peterson T. A. Ruskin J. N. Peterson R. T. MacRae C. A. (2003). Drugs that induce repolarization abnormalities cause bradycardia in zebrafish. *Circulation* 107 1355–1358. 10.1161/01.cir.0000061912.88753.87 12642353

[B107] Miura G. I. Yelon D. (2011). A guide to analysis of cardiac phenotypes in the zebrafish embryo. *Methods Cell. Biol.* 101 161–180. 10.1016/B978-0-12-387036-0.00007-4 21550443 PMC3292854

[B108] Mochizuki T. Wu G. Hayashi T. Xenophontos S. L. Veldhuisen B. Saris J. J. (1996). PKD2, a gene for polycystic kidney disease that encodes an integral membrane protein. *Science* 272 1339–1342. 10.1126/science.272.5266.1339 8650545

[B109] Nagata Y. Yamagishi M. Konno T. Nakanishi C. Asano Y. Ito S. (2017). Heat failure phenotypes induced by knockdown of DAPIT in zebrafish: a new insight into mechanism of dilated cardiomyopathy. *Sci. Rep.* 7:17417. 10.1038/s41598-017-17572-y 29234032 PMC5727169

[B110] Nakagama Y. Takeda N. Ogawa S. Takeda H. Furutani Y. Nakanishi T. (2020). Noonan syndrome-associated biallelic LZTR1 mutations cause cardiac hypertrophy and vascular malformations in zebrafish. *Mol. Genet. Genomic Med.* 8:e1107. 10.1002/mgg3.1107 31883238 PMC7057116

[B111] Nakamura M. Sadoshima J. (2018). Mechanisms of physiological and pathological cardiac hypertrophy. *Nat. Rev. Cardiol.* 15 387–407. 10.1038/s41569-018-0007-y 29674714

[B112] Nakayama H. Chen X. Baines C. P. Klevitsky R. Zhang X. Zhang H. (2007). Ca2+- and mitochondrial-dependent cardiomyocyte necrosis as a primary mediator of heart failure. *J. Clin. Invest.* 117 2431–2444. 10.1172/JCI31060 17694179 PMC1937500

[B113] Narumanchi S. Kalervo K. Perttunen S. Wang H. Immonen K. Kosonen R. (2019). Inhibition of let-7c regulates cardiac regeneration after cryoinjury in adult zebrafish. *J. Cardiovasc. Dev. Dis.* 6:16. 10.3390/jcdd6020016 30987331 PMC6617397

[B114] Nasevicius A. Ekker S. C. (2000). Effective targeted gene ‘knockdown’ in zebrafish. *Nat. Genet.* 26 216–220. 10.1038/79951 11017081

[B115] Nemtsas P. Wettwer E. Christ T. Weidinger G. Ravens U. (2010). Adult zebrafish heart as a model for human heart? An electrophysiological study. *J. Mol. Cell Cardiol.* 48 161–171. 10.1016/j.yjmcc.2009.08.034 19747484

[B116] Norman T. D. Mc B. R. (1958). Cardiac hypertrophy in rats with phenylhydrazine anemia. *Circ. Res.* 6 765–770. 10.1161/01.res.6.6.765 13585605

[B117] Norton N. Li D. Rieder M. J. Siegfried J. D. Rampersaud E. Zuchner S. (2011). Genome-wide studies of copy number variation and exome sequencing identify rare variants in BAG3 as a cause of dilated cardiomyopathy. *Am. J. Hum. Genet.* 88 273–282. 10.1016/j.ajhg.2011.01.016 21353195 PMC3059419

[B118] Orr N. Arnaout R. Gula L. J. Spears D. A. Leong-Sit P. Li Q. (2016). A mutation in the atrial-specific myosin light chain gene (MYL4) causes familial atrial fibrillation. *Nat. Commun.* 7:11303. 10.1038/ncomms11303 27066836 PMC4832069

[B119] Paavola J. Alakoski T. Ulvila J. Kilpiö T. Sirén J. Perttunen S. (2020). Vezf1 regulates cardiac structure and contractile function. *EBioMedicine* 51:102608. 10.1016/j.ebiom.2019.102608 31911272 PMC6948172

[B120] Paavola J. Schliffke S. Rossetti S. Kuo I. Y. Yuan S. Sun Z. (2013). Polycystin-2 mutations lead to impaired calcium cycling in the heart and predispose to dilated cardiomyopathy. *J. Mol. Cell Cardiol.* 58 199–208. 10.1016/j.yjmcc.2013.01.015 23376035 PMC3636149

[B121] Parente V. Balasso S. Pompilio G. Verduci L. Colombo G. I. Milano G. (2013). Hypoxia/reoxygenation cardiac injury and regeneration in zebrafish adult heart. *PLoS One* 8:e53748. 10.1371/journal.pone.0053748 23341992 PMC3547061

[B122] Pieske B. Trost S. Schutt K. Minami K. Just H. Hasenfuss G. (1998). Influence of forskolin on the force-frequency behavior in nonfailing and end-stage failing human myocardium. *Basic Res. Cardiol.* 93(Suppl. 1), 66–75. 10.1007/s003950050222 9833133

[B123] Pogwizd S. M. Bers D. M. (2002). Calcium cycling in heart failure: the arrhythmia connection. *J. Cardiovasc. Electrophysiol.* 13 88–91. 10.1046/j.1540-8167.2002.00088.x 11843491

[B124] Ponikowski P. Voors A. A. Anker S. D. Bueno H. Cleland J. G. F. Coats A. J. S. (2016). 2016 ESC Guidelines for the diagnosis and treatment of acute and chronic heart failure: the Task Force for the diagnosis and treatment of acute and chronic heart failure of the European Society of Cardiology (ESC)Developed with the special contribution of the Heart Failure Association (HFA) of the ESC. *Eur. Heart J.* 37 2129–2200. 10.1093/eurheartj/ehw128 27206819

[B125] Poss K. D. Wilson L. G. Keating M. T. (2002). Heart regeneration in zebrafish. *Science* 298 2188–2190. 10.1126/science.1077857 12481136

[B126] Rath N. Wang Z. Lu M. M. Morrisey E. E. (2005). LMCD1/Dyxin is a novel transcriptional cofactor that restricts GATA6 function by inhibiting DNA binding. *Mol. Cell. Biol.* 25 8864–8873. 10.1128/MCB.25.20.8864-8873.2005 16199866 PMC1265795

[B127] Reischauer S. Arnaout R. Ramadass R. Stainier D. Y. (2014). Actin binding GFP allows 4D in vivo imaging of myofilament dynamics in the zebrafish heart and the identification of Erbb2 signaling as a remodeling factor of myofibril architecture. *Circ. Res.* 115 845–856. 10.1161/CIRCRESAHA.115.304356 25228389 PMC4371144

[B128] Rockman H. A. Wachhorst S. P. Mao L. Ross J. Jr. (1994). ANG II receptor blockade prevents ventricular hypertrophy and ANF gene expression with pressure overload in mice. *Am. J. Physiol.* 266(6 Pt 2), H2468–H2475. 10.1152/ajpheart.1994.266.6.H2468 8024008

[B129] Romano N. Ceci M. (2020). Are microRNAs responsible for cardiac hypertrophy in fish and mammals? What we can learn in the activation process in a zebrafish ex vivo model. *Biochim. Biophys. Acta Mol. Basis Dis.* 1866:165896. 10.1016/j.bbadis.2020.165896 32681863

[B130] Rovira M. Borras D. M. Marques I. J. Puig C. Planas J. V. (2018). Physiological responses to swimming-induced exercise in the adult zebrafish regenerating heart. *Front. Physiol.* 9:1362. 10.3389/fphys.2018.01362 30327615 PMC6174316

[B131] Sanchez-Iranzo H. Galardi-Castilla M. Sanz-Morejon A. Gonzalez-Rosa J. M. Costa R. Ernst A. (2018). Transient fibrosis resolves via fibroblast inactivation in the regenerating zebrafish heart. *Proc. Natl. Acad. Sci. U.S.A.* 115 4188–4193. 10.1073/pnas.1716713115 29610343 PMC5910827

[B132] Sander J. D. Cade L. Khayter C. Reyon D. Peterson R. T. Joung J. K. (2011). Targeted gene disruption in somatic zebrafish cells using engineered TALENs. *Nat. Biotechnol.* 29 697–698. 10.1038/nbt.1934 21822241 PMC3154023

[B133] Santoso F. Farhan A. Castillo A. L. Malhotra N. Saputra F. Kurnia K. A. (2020). An overview of methods for cardiac rhythm detection in Zebrafish. *Biomedicines* 8:329. 10.3390/biomedicines8090329 32899676 PMC7554775

[B134] Sanz-Morejon A. Mercader N. (2020). Recent insights into zebrafish cardiac regeneration. *Curr. Opin. Genet. Dev.* 64 37–43. 10.1016/j.gde.2020.05.020 32599303

[B135] Sarantis P. Gaitanaki C. Beis D. (2019). Ventricular remodeling of single-chambered myh6(-/-) adult zebrafish hearts occurs via a hyperplastic response and is accompanied by elastin deposition in the atrium. *Cell Tissue Res.* 378 279–288. 10.1007/s00441-019-03044-4 31129720

[B136] Sato M. Yost H. J. (2003). Cardiac neural crest contributes to cardiomyogenesis in zebrafish. *Dev. Biol.* 257 127–139. 10.1016/s0012-1606(03)00037-x 12710962

[B137] Scheid L. M. Mosqueira M. Hein S. Kossack M. Juergensen L. Mueller M. (2016). Essential light chain S195 phosphorylation is required for cardiac adaptation under physical stress. *Cardiovasc. Res.* 111 44–55. 10.1093/cvr/cvw066 27013636

[B138] Schnabel K. Wu C. C. Kurth T. Weidinger G. (2011). Regeneration of cryoinjury induced necrotic heart lesions in zebrafish is associated with epicardial activation and cardiomyocyte proliferation. *PLoS One* 6:e18503. 10.1371/journal.pone.0018503 21533269 PMC3075262

[B139] Schulte-Merker S. Stainier D. Y. (2014). Out with the old, in with the new: reassessing morpholino knockdowns in light of genome editing technology. *Development* 141 3103–3104. 10.1242/dev.112003 25100652

[B140] Sehnert A. J. Huq A. Weinstein B. M. Walker C. Fishman M. Stainier D. Y. (2002). Cardiac troponin T is essential in sarcomere assembly and cardiac contractility. *Nat. Genet.* 31 106–110. 10.1038/ng875 11967535

[B141] Shah A. N. Davey C. F. Whitebirch A. C. Miller A. C. Moens C. B. (2015). Rapid reverse genetic screening using CRISPR in zebrafish. *Nat. Methods* 12 535–540. 10.1038/nmeth.3360 25867848 PMC4667794

[B142] Shi X. Verma S. Yun J. Brand-Arzamendi K. Singh K. K. Liu X. (2017). Effect of empagliflozin on cardiac biomarkers in a zebrafish model of heart failure: clues to the EMPA-REG OUTCOME trial? *Mol. Cell Biochem.* 433 97–102. 10.1007/s11010-017-3018-9 28391552

[B143] Shi X. Zhang Y. Chen R. Gong Y. Zhang M. Guan R. (2020a). ndufa7 plays a critical role in cardiac hypertrophy. *J. Cell Mol. Med.* 24 13151–13162. 10.1111/jcmm.15921 32989924 PMC7701565

[B144] Shi X. Zhang Y. Gong Y. Chen M. Brand-Arzamendi K. Liu X. (2020b). Zebrafish hhatla is involved in cardiac hypertrophy. *J. Cell Physiol.* 236 3700–3709. 10.1002/jcp.30106 33052609

[B145] Shih Y. H. Zhang Y. Ding Y. Ross C. A. Li H. Olson T. M. (2015). Cardiac transcriptome and dilated cardiomyopathy genes in zebrafish. *Circ. Cardiovasc. Genet.* 8 261–269. 10.1161/CIRCGENETICS.114.000702 25583992 PMC4406804

[B146] Simoes F. C. Cahill T. J. Kenyon A. Gavriouchkina D. Vieira J. M. Sun X. (2020). Macrophages directly contribute collagen to scar formation during zebrafish heart regeneration and mouse heart repair. *Nat. Commun.* 11:600. 10.1038/s41467-019-14263-2 32001677 PMC6992796

[B147] Stainier D. Y. R. Raz E. Lawson N. D. Ekker S. C. Burdine R. D. Eisen J. S. (2017). Guidelines for morpholino use in zebrafish. *PLoS Genet.* 13:e1007000. 10.1371/journal.pgen.1007000 29049395 PMC5648102

[B148] Sun J. She P. Liu X. Gao B. Jin D. Zhong T. P. (2020). Disruption of Abcc6 transporter in zebrafish causes ocular calcification and cardiac fibrosis. *Int. J. Mol. Sci.* 22:278. 10.3390/ijms22010278 33383974 PMC7795442

[B149] Sun X. Hoage T. Bai P. Ding Y. Chen Z. Zhang R. (2009). Cardiac hypertrophy involves both myocyte hypertrophy and hyperplasia in anemic zebrafish. *PLoS One* 4:e6596. 10.1371/journal.pone.0006596 19672293 PMC2719798

[B150] Sun Y. Wang Q. Fang Y. Wu C. Lu G. Chen Z. (2017). Activation of the Nkx2.5-Calr-p53 signaling pathway by hyperglycemia induces cardiac remodeling and dysfunction in adult zebrafish. *Dis. Model. Mech.* 10 1217–1227. 10.1242/dmm.026781 28801532 PMC5665450

[B151] Tham Y. K. Bernardo B. C. Ooi J. Y. Weeks K. L. McMullen J. R. (2015). Pathophysiology of cardiac hypertrophy and heart failure: signaling pathways and novel therapeutic targets. *Arch. Toxicol.* 89 1401–1438. 10.1007/s00204-015-1477-x 25708889

[B152] Thierfelder L. Watkins H. MacRae C. Lamas R. McKenna W. Vosberg H. P. (1994). Alpha-tropomyosin and cardiac troponin T mutations cause familial hypertrophic cardiomyopathy: a disease of the sarcomere. *Cell* 77 701–712. 10.1016/0092-8674(94)90054-x 8205619

[B153] Thorsen K. Dam V. S. Kjaer-Sorensen K. Pedersen L. N. Skeberdis V. A. Jurevicius J. (2017). Loss-of-activity-mutation in the cardiac chloride-bicarbonate exchanger AE3 causes short QT syndrome. *Nat. Commun.* 8:1696. 10.1038/s41467-017-01630-0 29167417 PMC5700076

[B154] Tomaselli G. F. Beuckelmann D. J. Calkins H. G. Berger R. D. Kessler P. D. Lawrence J. H. (1994). Sudden cardiac death in heart failure. The role of abnormal repolarization. *Circulation* 90 2534–2539. 10.1161/01.cir.90.5.2534 7955213

[B155] Van B. Nishi M. Komazaki S. Ichimura A. Kakizawa S. Nakanaga K. (2015). Mitsugumin 56 (hedgehog acyltransferase-like) is a sarcoplasmic reticulum-resident protein essential for postnatal muscle maturation. *FEBS Lett.* 589 1095–1104. 10.1016/j.febslet.2015.03.028 25841338

[B156] Verkerk A. O. Remme C. A. (2012). Zebrafish: a novel research tool for cardiac (patho)electrophysiology and ion channel disorders. *Front. Physiol.* 3:255. 10.3389/fphys.2012.00255 22934012 PMC3429032

[B157] Virani S. S. Alonso A. Benjamin E. J. Bittencourt M. S. Callaway C. W. Carson A. P. (2020). Heart disease and stroke statistics-2020 update: a report from the american heart association. *Circulation* 141 e139–e596. 10.1161/CIR.0000000000000757 31992061

[B158] Vornanen M. Hassinen M. (2016). Zebrafish heart as a model for human cardiac electrophysiology. *Channels* 10 101–110. 10.1080/19336950.2015.1121335 26671745 PMC4960994

[B159] Wang J. Huertas-Vazquez A. Wang Y. Lusis A. J. (2019). Isoproterenol-induced cardiac diastolic dysfunction in mice: a systems genetics analysis. *Front. Cardiovasc. Med.* 6:100. 10.3389/fcvm.2019.00100 31417910 PMC6684968

[B160] Wang J. Panakova D. Kikuchi K. Holdway J. E. Gemberling M. Burris J. S. (2011). The regenerative capacity of zebrafish reverses cardiac failure caused by genetic cardiomyocyte depletion. *Development* 138 3421–3430. 10.1242/dev.068601 21752928 PMC3143562

[B161] Wang L. W. Huttner I. G. Santiago C. F. Kesteven S. H. Yu Z. Y. Feneley M. P. (2017). Standardized echocardiographic assessment of cardiac function in normal adult zebrafish and heart disease models. *Dis. Model. Mech.* 10 63–76. 10.1242/dmm.026989 28067629 PMC5278526

[B162] Wang Q. Luo C. Lu G. Chen Z. (2020). Effect of adenosine monophosphate-activated protein kinase-p53-Kruppel-like factor 2a pathway in hyperglycemia-induced cardiac remodeling in adult zebrafish. *J. Diabetes Investig.* 12 320–333. 10.1111/jdi.13393 32881390 PMC7926222

[B163] Wu Y. Rasmussen T. P. Koval O. M. Joiner M. L. Hall D. D. Chen B. (2015). The mitochondrial uniporter controls fight or flight heart rate increases. *Nat. Commun.* 6:6081. 10.1038/ncomms7081 25603276 PMC4398998

[B164] Xu J. Li Z. Ren X. Dong M. Li J. Shi X. (2015). Investigation of pathogenic genes in chinese sporadic hypertrophic cardiomyopathy patients by whole exome sequencing. *Sci. Rep.* 5:16609. 10.1038/srep16609 26573135 PMC4647833

[B165] Xu X. Meiler S. E. Zhong T. P. Mohideen M. Crossley D. A. Burggren W. W. (2002). Cardiomyopathy in zebrafish due to mutation in an alternatively spliced exon of titin. *Nat. Genet.* 30 205–209. 10.1038/ng816 11788825

[B166] Zakaria Z. Z. Benslimane F. M. Nasrallah G. K. Shurbaji S. Younes N. N. Mraiche F. (2018). Using zebrafish for investigating the molecular mechanisms of drug-induced cardiotoxicity. *Biomed. Res. Int.* 2018:1642684. 10.1155/2018/1642684 30363733 PMC6180974

[B167] Zang L. Maddison L. A. Chen W. (2018). Zebrafish as a model for obesity and diabetes. *Front. Cell Dev. Biol.* 6:91. 10.3389/fcell.2018.00091 30177968 PMC6110173

[B168] Zhou Z. Zheng L. Tang C. Chen Z. Zhu R. Peng X. (2020). Identification of potentially relevant genes for excessive exercise-induced pathological cardiac hypertrophy in zebrafish. *Front. Physiol.* 11:565307. 10.3389/fphys.2020.565307 33329019 PMC7734032

[B169] Zhu J. J. Xu Y. Q. He J. H. Yu H. P. Huang C. J. Gao J. M. (2014). Human cardiotoxic drugs delivered by soaking and microinjection induce cardiovascular toxicity in zebrafish. *J. Appl. Toxicol.* 34 139–148. 10.1002/jat.2843 23307606

[B170] Zhu X. Y. Wu S. Q. Guo S. Y. Yang H. Xia B. Li P. (2018). A zebrafish heart failure model for assessing therapeutic agents. *Zebrafish* 15 243–253. 10.1089/zeb.2017.1546 29653073

